# Myelin-reactive CD8^+^ T cells influence conventional dendritic cell subsets towards a mature and regulatory phenotype in experimental autoimmune encephalomyelitis

**DOI:** 10.1186/s12974-025-03377-8

**Published:** 2025-02-28

**Authors:** Mohit Upadhye, Connor R. Wilhelm, Kai J. Rogers, Chakrapani Vemulawada, Nicholas Borcherding, Alexander W. Boyden, Kevin L. Legge, Nitin J. Karandikar

**Affiliations:** 1 Department of Pathology, University of Iowa Health Care, 200 Hawkins Drive, C660 GH, Iowa City, IA 52242 USA; 2 Iowa City Veterans Affairs Medical Center, Iowa City, IA 52246 USA; 3 Department of Pathology and Immunology, Washington University in St. Louis, 660 S. Euclid Ave, St. Louis, MO 63110 USA

## Abstract

Multiple sclerosis (MS), an autoimmune demyelinating disease of the central nervous system, is modeled in mice as experimental autoimmune encephalomyelitis (EAE). While CD4^+^ T cells, primarily Th1 and Th17 subsets, drive disease pathogenesis, the exact function of CD8^+^ T cells remains unclear. We previously demonstrated that adoptively transferred myelin-reactive CD8^+^ T cells (PLP-CD8) prevent EAE induction and suppress ongoing disease through the engagement of MHC Class-I in recipient mice. Here, we show that PLP-CD8 induce regulatory changes in both subsets of conventional dendritic cells (cDC1 and CD11b^+^ cDC) in vivo and in vitro. Adoptively transferred PLP-CD8 promoted both cDC subsets to adopt a mature and regulatory phenotype with an anti-inflammatory cytokine profile and a reduced capacity to support CD4^+^ T cell proliferation. In vitro, PLP-CD8 induced similar phenotypic changes in both cDC subsets in an antigen-specific, dose-dependent manner. PLP-CD8 directly interacted with cDC1 and indirectly influenced CD11b^+^ cDC through paracrine signaling. Notably, direct interaction with PLP-CD8 had detrimental effects on CD11b^+^ cDC. Single-cell RNA sequencing revealed upregulation of key immunoregulatory genes, such as *Foxo3*, in both cDC subsets with enrichment of pathways involved in immune regulation and T cell differentiation. Our study highlights a novel mechanism in which myelin-reactive CD8^+^ T cells directly interact with cDC1 and modulate CD11b^+^ cDC through paracrine mechanisms to induce mature, regulatory dendritic cells, which leads to inhibited CD4^+^ T cell responses and reduced EAE pathogenesis.

**Supplementary Information:**

The online version contains supplementary material available at 10.1186/s12974-025-03377-8.

## Introduction

Multiple sclerosis (MS) is an autoimmune demyelinating disease of the central nervous system (CNS) that predominantly affects young adults, leading to significant neurological disability [1–3]. With nearly 1 million cases in the United States alone, MS is more prevalent in developed countries and urbanized regions of the developing world [1, 4].

The etiopathogenesis of MS is complex, involving a multifactorial autoimmune response directed against myelin antigens, largely mediated by autoreactive T cells [5, 6]. This autoimmune process is commonly studied using the rodent model, experimental autoimmune encephalomyelitis (EAE) [7, 8]. CD4^+^ T cells, particularly the Th1 and Th17 subsets, are critical in driving EAE pathogenesis [9–11]. CD8^+^ T cells are more abundant in human MS lesions and are traditionally viewed as pathogenic due to their cytotoxic potential [12, 13]. However, the precise role of CD8^+^ T cells in MS and EAE remains unclear.

Our previous work demonstrated that myelin-reactive CD8^+^ + T cells may possess autoregulatory properties in humans and mice (reviewed in [14–16]). Specifically, CD8^+^ T cells from healthy human donors as well MS patients during disease quiescence can suppress CD4^+^ T cell proliferation in vitro, whereas CD8^+^ T cells during disease exacerbation show impaired suppressive capacity [17–19]. In mice, the adoptive transfer of myelin-reactive CD8^+^ T cells inhibits EAE severity and ameliorates ongoing disease, highlighting their protective potential [20–25]. Our previous findings suggest an association between this CD8^+^ T cell-mediated protection and functional changes in splenic dendritic cells (DC) and attenuation of CD4^+^ T cell responses in mice [26].

As key antigen-presenting cells (APC), DC initiate and regulate adaptive T-cell responses [27–29]. DC subsets include plasmacytoid DC (pDC) and conventional DC (cDC), with cDC further divided into two major subsets – cDC1 (CD8^+^, XCR-1^+^, CD11b^−^, CD172a^−^) and cDC2 (CD8^−^, XCR-1^−^, CD11b^+^, CD172a^+^) [30–32]. pDC primarily drive antiviral T-cell responses by producing large amounts of type I interferons [33]. cDC1 are uniquely equipped for cross-presenting extracellular antigens to CD8^+^ T cells via MHC-I, a process facilitated by their specialized antigen processing machinery and high expression of cross-presentation-related molecules [34–36]. Additionally, cDC1 promote the differentiation of CD4^+^ T cells into Th1 cells, which are pivotal for initiating pro-inflammatory immune responses [37]. Conversely, cDC2 are highly effective at presenting antigens to CD4^+^ T cells, skewing their differentiation towards Th2 and Th17 cells, with the latter playing a central role in driving the pathogenesis of experimental autoimmune encephalomyelitis (EAE) [38, 39]. cDC subsets also engage in cytokine-driven crosstalk, often with CD4^+^ T cells acting as intermediaries [40, 41]. Additionally, immature DC induce anergy in autoreactive T cells and maintain tolerance to autoantigens [42, 43]. Notably, several other DC subpopulations such as monocyte-derived DC (moDC) and inflammatory DC also express CD11b and CD172a and are phenotypically similar to cDC2 [44, 45]. These populations can be further differentiated using additional markers.

Given the pivotal role of DC-T cell interactions in immune regulation, and building on our previous findings, this study investigated the hypotheses that adoptively transferred myelin-reactive CD8^+^ T cells induce the differentiation of regulatory DC in recipient mice and that these regulatory DC, in turn, modulate pathogenic CD4^+^ T cell responses, thereby reducing disease. To test this hypothesis, we conducted a comprehensive analysis of the functional and phenotypic changes within various DC subsets upon interaction with myelin-reactive CD8^+^ T cells. Additionally, we sought to recapitulate these interactions using in vitro assays to elucidate the specific effects of CD8^+^ T cells on individual cDC subsets. This study uncovers the mechanisms by which myelin-reactive CD8^+^ T cells induce regulatory DC and suppress pathogenic CD4^+^ T cell responses. These findings enhance our understanding of DC-mediated immune regulation and provide insights into its therapeutic potential for autoimmune diseases such as MS.

## Materials and methods

### Mice

All experiments were performed using 8–10-week-old female wild-type C57BL/6 J (WT B6) mice were purchased from Jackson Laboratories (Bar Harbor, ME). Mice were housed in barrier rooms at the University of Iowa Animal Care Facility, were subjected to a 12-h light/dark cycle. Mice had unrestricted access to food and water and were cared for humanely, adhering to the guidelines set by the University of Iowa Institutional Animal Care and Use Committee and the NIH Guide for the Care and Use of Laboratory Animals (NIH Publication No. 8023, revised 1978).

### Peptides

PLP_178-191_ (NTWTTCQSIAFPSK) and OVA_323-339_ (ISQAVHAAHAEINEAGR) were purchased from GenScript (Piscataway, NJ).

### Immunizations and EAE induction

Mice were immunized subcutaneously in the flanks with 100 µg of PLP_178-191_ or OVA_323-339_ emulsified in Complete Freund’s Adjuvant (CFA) supplemented with 4 mg/ml Mycobacterium tuberculosis (Becton Dickinson, Franklin Lakes, NJ). The day of immunization was considered day 0. For EAE induction, mice immunized with PLP_178-191_ received two doses of 250 ng of Pertussis Toxin (PTX) in glycerol (List Labs, California) on days 0 and 2 intraperitoneally. The mice were observed for ascending paralysis until day 25. The disease was scored as 0 for no symptoms; 1 for tail weakness; 2 for mild hindlimb weakness but no paralysis; 3 for partial hindlimb paralysis; 4 for complete hindlimb paralysis and 5 for moribund state or death.

### Enrichment and adoptive transfer of myelin-reactive CD8^+^ T cells

Mice were immunized with the myelin peptide, PLP_178-191_ or the control peptide, OVA_323-339_ on day 0. Spleens and inguinal lymph nodes were harvested from immunized mice between days 15 to 20 and processed into single-cell suspensions. Cells were cultured at 7 × 10^6^ cells/ml in the presence of 20 µg/ml of cognate peptide (PLP_178-191_ or OVA_323-339_) and 10 pg/ml of rIL-2 at 37ºC for 72 h following which CD8^+^ T cells were enriched by magnetic bead sort (Miltenyi Biotech, Auburn, CA) with a purity of > 95%. In vitro-activated CD8^+^ T cells enriched from PLP-immunized mice were called PLP-CD8 and those from OVA-immunized mice were called OVA-CD8. For the adoptive transfer experiments, 5 × 10^6^ CD8^+^ T cells (PLP-CD8 or OVA-CD8) were injected intravenously into each recipient mouse on day -1 and EAE was induced on day 0 as described above.

### Preparation of Nycodenz working solution

Nycodenz stock solution was prepared by dissolving 15.275 g Nycodenz (Serumwerk) in 50 ml MilliQ water and stored at 4 ℃. Nycodenz buffer was prepared by mixing 9 g NaCl, 0.6055 g Tris, 0.22368 g KCl and 0.11167 g EDTA in 1.12L of MilliQ water. Nycodenz working solution was prepared by mixing 54% Nycodenz buffer and 46% Nycodenz stock solution for the desired volume.

### Isolation of DC

Spleens were harvested from PLP_178-191_-immunized mice at specific time-points and homogenized in complete media containing 200 U/ml of collagenase (Worthington Type 3) and 10 mM EDTA (Invitrogen). The homogenates were incubated at 37 ℃ for 30 min with gentle agitation to ensure enzymatic digestion and subsequently filtered to obtain single-cell suspensions. Antigen-presenting cells were enriched using Nycodenz gradient centrifugation. Briefly, cells were washed in RPMI to remove residual enzymes and resuspended in 5 ml of Nycodenz working solution (prepared as described above). A 5-ml overlay of 1% HBSS was carefully added to the Nycodenz-cell suspension to create a density gradient. The gradient was centrifuged at 1800 × g for 15 min at room temperature with the brake off to preserve the gradient layers. The interface containing enriched antigen-presenting cells was carefully collected, and dendritic cells (DC) were further purified using CD11c MicroBeads UltraPure (Miltenyi Biotech, Auburn, CA). The final preparation yielded DC with > 90% purity, as confirmed by flow cytometry.

### DC-CD8 interaction in vitro

Splenic DC were isolated from PLP_178-191_-immunized mice between 10–12 days following immunization. PLP-CD8 and OVA-CD8 were enriched from immunized mice and activated in vitro as explained above. DC were incubated with in vitro-activated PLP or OVA-CD8 at increasing ratios from 1:1.25 to 1:5 (DC: CD8^+^) in the presence of PLP_178-191_ (20 µg/ml) and rIL-2 (10 pg/ml) for 24 h. Freshly isolated DC (direct ex vivo) and DC incubated without CD8^+^ T cells (No CD8^+^) served as controls. Following incubation, cells were washed and stained with Live-Dead and subsequently for surface markers and analyzed using flow cytometry.

### Trans-well assay

Splenic DC were isolated from PLP_178-191_-immunized mice on day 12 post-immunization. Conventional DC (cDC) were identified on a flow cytometer (Cytek Aurora, Fremont, CA) as CD11c^hi^, MHC-II^hi^ after excluding B cells (CD19^+^) and pDC (Siglec-H^+^ CD11b^−^). cDC subsets were further distinguished as cDC1 (XCR-1^+^ CD8^+^ CD172a^−^ CD11b^−^) and CD11b^+^ cDC (XCR-1^−^ CD8^−^ CD172a^+^ CD11b^+^) and sorted using Fluorescence-Activated Cell Sorting (FACS, Cytek). Markers, XCR-1 and CD172a, were selected over CD8 and CD11b for subset identification due to superior staining and delineation in FACS analyses. For phenotypic characterization, cDC subsets were defined as cDC1 (CD8^+^ CD11b^−^) and CD11b^+^ cDC (CD8^−^ CD11b ^+^) by flow cytometry. A trans-well setup was employed using 24-well tissue culture plates with 0.4-µm semipermeable membrane inserts (Millipore Sigma). CD11b^+^ cDC (80,000) were seeded into the lower wells, while cDC1 (20,000) into the inserts. PLP-CD8 (500,000), obtained from enrichment cultures, were incubated with either cDC1 or CD11b^+^ cDC in the presence of PLP_178-191_ and rIL-2 for 24 h following which supernatants were collected and cells were analyzed by flow cytometric.

### DC Cytokine analysis

Splenic DC were isolated from PLP or OVA-CD8^+^ recipients on days 3 and 13 post-EAE induction. 250,000 DC were stimulated with monoclonal anti-CD40 antibody (100 µg/ml, Clone: FGK4.5/FGK45, BioXCell) for 48 h in 200 µL media. Levels of IL-12p70 and IL-10 in the supernatants were measured by ELISA (R&D Systems, Quantikine ELISA kits).

### CD4^+^ T cell proliferation assay

CD4^+^ T cells were isolated from PLP_178-191_-immunized mice (12 days post-immunization) using magnetic bead sorting (CD4^+^ (L3T4) Miltenyi Biotech, Auburn, CA) and labelled with CFSE (0.25 mM) (Invitrogen) as responder CD4^+^ T cells. DC were isolated from PLP-CD8 recipients (PLP-CD8-DC) or OVA-CD8 recipients (OVA-CD8-DC) on day 12 post-EAE induction and cocultured with responder CD4^+^ T cells at 1:10 ratio (DC:CD4^+^) with rIL-2 (10 pg/ml) and increasing concentrations of PLP_178-191_ (0 to 50 µM) for 5 days. Proliferation of live CD4^+^ T cell proliferation was assessed by CFSE dilution using flow cytometry.

### Antibody staining for flow cytometric analysis

To assess cellular viability, cells were washed and briefly incubated with Live-Dead Blue (ThermoFisher) in PBS, following the manufacturer’s protocol. After viability staining, cells were incubated with Fc receptor-blocking antibody (BioLegend) and stained for surface markers using the following anti-mouse antibodies: Siglec-H (BUV 661), CD8a (Pacific Blue), CD172a (BV 421) and PD-L2 (BV 711) from BD Biosciences; CD45 (APC), TCR-β (APC-Cy7), MHC-II (BV 785), CD11b (PerCP-Cy5.5), XCR1 (BV 510), CD86 (PE-Cy5), PD-L1 (PE-Dazzle 594), and CD83 (BV 650) from BioLegend; and CD19 (Super Bright 600), CD11c (FITC), CD40 (PerCP-eFluor 710), CD80 (Alexa Fluor 700), and LAP (PE-Cy7) from Invitrogen. Following surface staining, the cells were fixed with 4% paraformaldehyde for 20 min at 4ºC and washed and responded in FACS buffer for phenotypic characterization by flow cytometry.

### Single-cell RNA-sequencing

Splenic DC were isolated from CD8 recipients, PLP-CD8-DC and OVA-CD8-DC, on day 12 post-EAE induction as described before. The enrichment procedure efficiently removes non-DC antigen presenting cells (APC) and other cell types. DC were strained through a 40-µm filter, fixed using the Cell Fixation Kit and processed with the Evercode Whole Transcriptome Mini Kit from Parse Biosciences (Seattle, Washington, USA) according to the manufacturer’s guidelines. Sub-libraries were evaluated for adequacy (Agilent BioAnalyzer RNA integrity number > 8) and sent for Next Generation Sequencing on the Illumina NovaSeq 6000 using 100 bp paired-end reads (Iowa Institute of Human Genetics, University of Iowa). Resulting FASTQ files were further processed using the Parse Biosciences pipeline to identify the barcodes, align the reads, and create a gene-cell count matrix. Downstream data analysis and visualization were performed using the R package Seurat (Version 5.1.0) [46]. Quality control thresholds were applied to retain cells with 200–1,250 detected genes and < 10% mitochondrial gene expression; cells failing these criteria were excluded from further analysis. Principal component analysis (PCA) was performed on the top 2,000 variable features identified using the FindVariableFeatures function. Seurat objects corresponding to DC isolated from PLP-CD8 and OVA-CD8 recipients were integrated into a single dataset using the FindIntegrationAnchors and IntegrateData functions. Nearest-neighbor graphs were constructed using the FindNeighbors function with k = 20 and 30 principal components as input. Clustering was conducted using the Louvain algorithm (FindClusters function) with a resolution parameter of 0.5. Uniform Manifold Approximation and Projection (UMAP) was used for visualization with default settings. Canonical gene expression was analyzed to annotate clusters.

### Differential expression and functional analyses

Cluster-specific differentially expressed genes (DEGs) between the DC isolated from PLP-CD8 or OVA-CD8 recipients were identified using the FindMarkers function, employing the Wilcoxon rank-sum test. Genes with an adjusted p-value < 0.05 were considered differentially expressed. For functional characterization, KEGG pathway enrichment analysis was performed using the clusterProfiler R package [47] with default parameters. To infer ligand-receptor interactions mediating cellular communication, NicheNet [48] was used with default settings. Regulatory activity of transcription factors and signaling pathways was analyzed using the decoupleR package [49], applying default parameters. Results from all analyses were visualized using standard plotting functions.

### Scientific rigor

Unless indicated otherwise, all experiments were repeated at least twice and the representative data from a single experiment are shown in the figures. The two exceptions to this are: (1) The single-cell RNA sequencing was performed in a single experiment, using splenic DC isolated from two mice per treatment condition (PLP-CD8 and OVA-CD8); and (2) The experiment involving addition of supernatants from trans-well assays was performed once and included 3 technical replicates.

### Artificial intelligence

Writing assistance was provided by OpenAI's ChatGPT to enhance clarity and professionalism in the manuscript.

### Statistical analysis

Expression of costimulatory and regulatory markers was quantified in geometric mean fluorescence intensities (gMFI) by flow cytometry and the relative expression (RE) was calculated by normalizing data to control conditions. Similarly, the cytokine levels measured by ELISA were normalized to OVA-CD8-DC. Frequencies of gated populations in flow cytometric analysis were considered without normalization. Statistical analyses were performed using appropriate tests based on experimental design. Comparisons between two groups were conducted using a two-tailed, unpaired Student’s t test. For experiments involving more than two groups, one-way ANOVA with multiple comparisons was used, while comparisons involving multiple factors between two groups were analyzed using two-way ANOVA with multiple comparisons. The surface marker expression data satisfied the tests for normal distribution and were analyzed using parametric analyses. EAE scores were analyzed using the nonparametric Kruskal–Wallis test. The differences were considered statistically significant with *P < 0.05, **P < 0.01, ***P < 0.001.

## Results

### Adoptively transferred PLP-CD8 induce functional changes in splenic DC

**Fig. 1:**
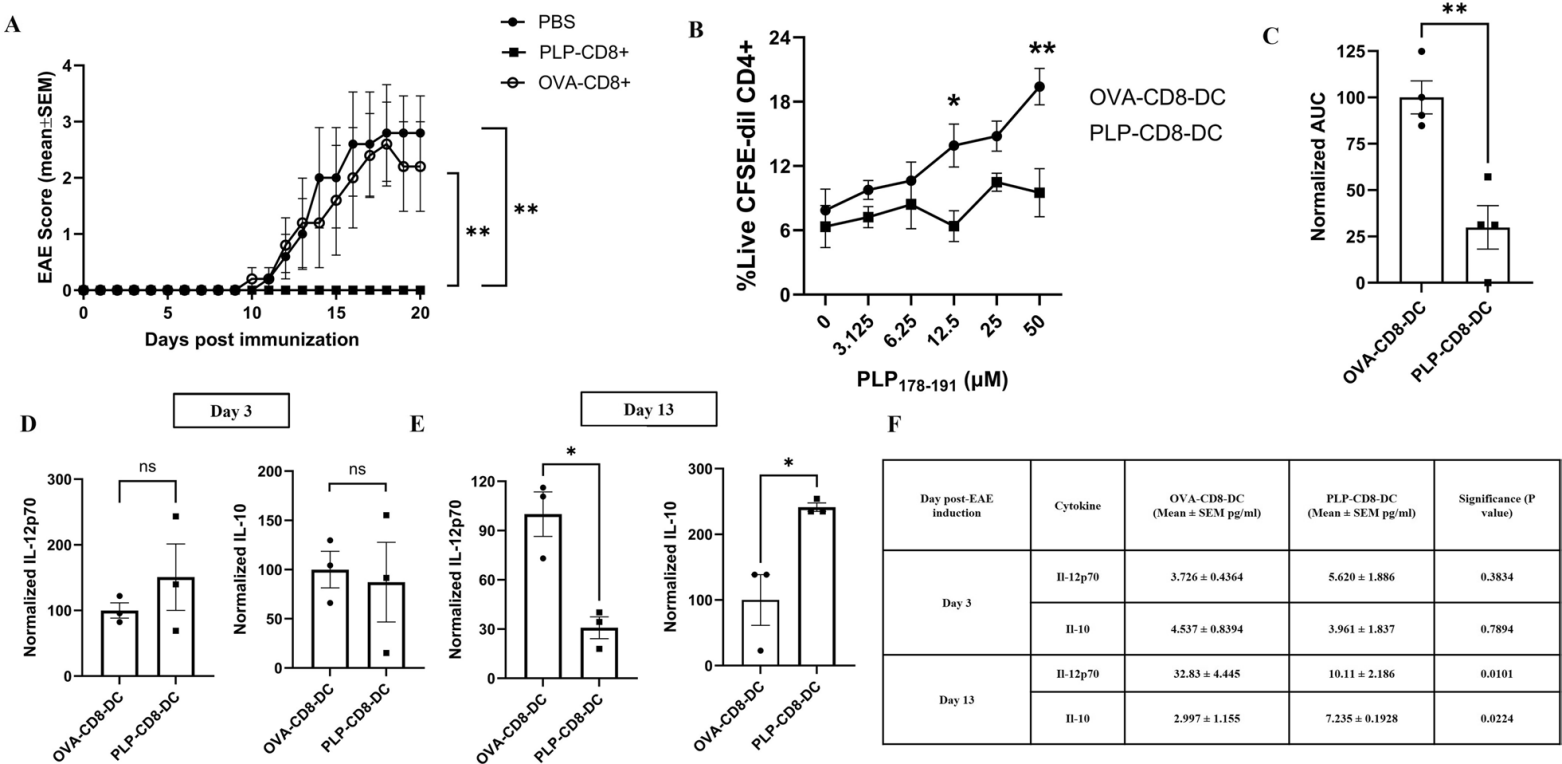
**PLP-CD8 induce functional changes in DC.** Five million CD8^+^ T cells reactive against the myelin peptide, PLP_178-191_ (PLP-CD8), or the control peptide, OVA_323-339_ (OVA-CD8), or PBS were adoptively transferred into wild-type recipient mice one day before inducing EAE with PLP_178-191_. **A** shows EAE scores (Mean ± SEM) of 3 groups of mice over 20 days post-EAE induction. Recipients of PBS or OVA-CD8 developed severe ascending paralysis while those of PLP-CD8 remained protected from EAE induction. (N = 5/group, Kruskal–Wallis test). **B**-**C** Splenic DC were isolated from PLP-CD8 recipients (PLP-CD8-DC) or OVA-CD8 recipients (OVA-CD8-DC) on day 12 post-EAE induction and cultured with CFSE-labelled CD4^+^ T cells from PLP-immunized mice at a 1:10 ratio (DC: CD4^+^) with rIL-2 (10 pg/ml) and increasing concentrations of PLP_178-191_ (0 to 50 µM) for 5 days. **B** CD4^+^ T cell proliferation (%CFSE-dilute CD4^+^) was plotted as a function of increasing peptide concentrations and was significantly higher with OVA-CD8-DC (N = 3, Two-way ANOVA with multiple comparisons). **C** The area under the curve (AUC) was calculated for the dose–response curves and data were normalized to OVA-CD8-DC. AUC for CD4.^+^ proliferation in response to increasing peptide concentrations was significantly lower with PLP-CD8-DC (N = 3, Student T-test). **D**-**F** PLP-CD8-DC and OVA-CD8-DC (250,000) isolated on days 3 and 13 post-EAE induction were stimulated with anti-CD40 antibody (100 µg/ml) for 48 h and the levels of IL-12p70 and IL-10 were measured in the supernatant using ELISA and normalized to OVA-CD8-DC. Normalized cytokine values on day 3 are shown in panel D on day 13 in panel E. Panel F shows the Mean ± SEM cytokine levels. Cytokine profile of both DC groups was similar on day 3 while PLP-CD8-DC produced lower levels of IL-12p70 and higher levels of IL-10 than OVA-CD8-DC on day 13 post-EAE induction (N = 3, student T-test) (ns = not significant, *p < 0.05, **p < 0.01)

We have previously demonstrated that CD8^+^ T cells reactive against myelin antigens such as MOG_35-55_, PLP-_178–191_, and MBP_84-104_ suppress EAE induced by their cognate antigen but not by another myelin antigen, suggesting an antigen-specific mechanism of EAE suppression in recipient mice [14, 20, 21, 23]. Adoptively transferred MOG_35-55_-reactive CD8^+^ T cells induced functional changes in the splenic dendritic cells (DC) of the recipient mice immunized with MOG_35-55_, but not in naïve/unimmunized mice or those immunized with control OVA_323-339_ antigen [26]. Building on these earlier findings, we hypothesized that myelin-reactive CD8^+^ T cells induce regulatory DC in the spleens of recipient mice, which subsequently attenuate pathogenic CD4^+^ T cell responses to prevent EAE induction. To test our hypothesis, we adoptively transferred 5 million *in-vitro*-activated CD8^+^ T cells from mice immunized with the myelin peptide PLP_178-191_ (PLP-CD8) or the control peptide OVA_323-339_ (OVA-CD8) or PBS into wild-type B6 mice one day before inducing EAE with PLP_178-191_ as described [22]. As expected, the recipients of PBS or OVA-CD8 developed severe ascending paralysis whereas PLP-CD8 recipients were protected from EAE induction (Fig. 1A, Supplementary Fig. 1). Splenic DC were isolated from PLP-CD8 recipients (PLP-CD8-DC) or OVA-CD8 recipients (OVA-CD8-DC) on day 12 post-EAE induction coinciding with the onset of EAE in controls (Supplementary Fig. 1).

CD4^+^ T cells were obtained 10 days post-immunization from a separate set of mice immunized with PLP_178-191._ These were stained with carboxyfluorescein succinimidyl ester (CFSE) to measure their proliferation. DC (PLP-CD8-DC or OVA-CD8-DC) were cocultured with CFSE-labelled CD4^+^ T cells at a 1:10 ratio (DC: CD4^+^) in the presence of rIL-2 and increasing concentrations of PLP_178-191_ for 5 days. CD4^+^ T cell proliferation was assessed by CFSE dilution at each peptide concentration. Supplementary Fig. 2 demonstrates representative flow plots of CFSE proliferation. Figure 1B shows cumulative data from a single representative experiment. The area under the curve (AUC) was calculated for both DC groups and data were normalized to OVA-CD8-DC (Fig. 1C). We observed significantly lower proliferation of CD4^+^ T cells with PLP-CD8-DC than with OVA-CD8-DC (Figs. 1B, C).

Separately, PLP-CD8-DC and OVA-CD8-DC were isolated on days 3 and 13 post-EAE induction, with day 3 chosen to detect potential early changes. DC were stimulated with monoclonal anti-CD40 antibody for 48 h, and supernatant levels of IL-12p70 (proinflammatory) and IL-10 (anti-inflammatory) were measured by ELISA and normalized to OVA-CD8-DC. While both DC groups exhibited similar cytokine profiles on day 3 (Figs. 1D, F), by day 13, PLP-CD8-DC produced significantly less IL-12p70 and more IL-10 than OVA-CD8-DC (Figs. 1E, F). These results suggest that PLP-CD8-DC undergo functional changes, marked by a diminished ability to support CD4^+^ T cell proliferation and the development of an anti-inflammatory cytokine profile following EAE induction.

### PLP-CD8-DC subsets display a mature and regulatory phenotype ex vivo

**Fig. 2:**
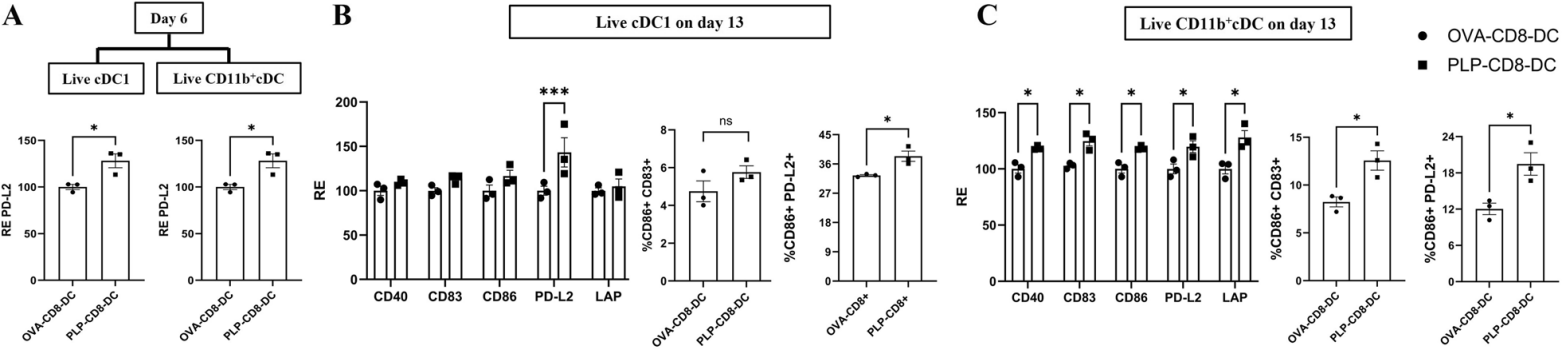
**PLP-CD8-DC exhibit mature and regulatory phenotype post-EAE induction.** PLP or OVA-CD8 were transferred one day before EAE-induction in the recipient mice. PLP-CD8-DC and OVA-CD8-DC were isolated on days 1, 3, 6 and 13 post-EAE induction, and stained with Live-Dead Blue and surface markers for the phenotypic characterization of conventional DC (cDC) subsets using flow cytometry. Expressions of costimulatory and regulatory markers on live cDC subsets were quantified with geometric mean fluorescence intensity (gMFI) and plotted as the relative expression (RE) normalized to OVA-CD8-DC. **A** By day 6 post-EAE induction, we observed PD-L2 upregulation on both cDC1 and CD11b^+^ cDC subsets of PLP-CD8-DC. **B** By day 13 post-EAE induction, the cDC1 subset of PLP-CD8-DC showed higher expression of PD-L2 and enrichment of CD86^+^ PD-L2^+^ population. **C** The CD11b^+^ cDC subset of PLP-CD8-DC displayed upregulation of all costimulatory and regulatory markers and higher co-expression of CD86 with CD83 and PD-L2 (N = 3, Two-Way ANOVA with multiple comparisons, T-test, ns = not significant, *p < 0.05, **p < 0.01, ***p < 0.001)

To investigate possible phenotypic changes in splenic DC from CD8 recipients, PLP-CD8-DC and OVA-CD8-DC were isolated on days 1, 3, 6, and 13 post-EAE induction. DC were stained with Live-Dead blue and subsequently for surface markers and analyzed using flow cytometry. Conventional DC (cDC) are the predominant subset of splenic DC involved in adaptive T cell response in EAE. Following the exclusion of non-DC cell types and pDC, cDC were identified as CD11c^hi^ MHC-Class II^hi^ and subdivided into cDC1 (CD8a^+^ CD11b^−^) and CD11b^+^ cDC (CD8a^−^ CD11b^+^) subsets. The cDC1 subset shows a higher expression of XCR-1 while CD11b^+^ cDC subset shows a higher expression of CD172a (Supplementary Fig. 3). Similar gating strategy was used for other experiments with the addition of the markers, XCR-1 and CD172a preferentially expressed on cDC1 and CD11b^+^ cDC, respectively (Supplementary Fig. 3). Expression of costimulatory markers (CD40, CD80, CD83, CD86) and inhibitory markers (PD-L1, PD-L2, and Latency-Associated Peptide (LAP), a marker of latent TGF-β) on live cDC subsets was assessed with geometric mean fluorescence intensity (gMFI) and the relative expression (RE) of these markers normalized to OVA-CD8-DC was compared for statistical analysis. Both DC groups expressed a similar phenotype on days 1 and 3 post-EAE induction (data not shown). By day 6, however, the levels of PD-L2 were significantly higher on both cDC subsets (cDC1 and CD11b^+^ cDC) of PLP-CD8-DC (Fig. 2A). Major phenotypic differences in PLP-CD8-DC were observed by day 13 post-immunization. cDC1 subset of PLP-CD8-DC showed a significantly higher expression of PD-L2 and its co-expression with CD86 (relative expression shown in Fig. 2B, raw gMFI values and representative pseudocolor plots shown in Supplementary Figs. 4 and 5, respectively). cDC1 also expressed higher levels of CD83, which were statistically significant when compared individually in gMFI values (Supplementary Fig. 4) but failed to reach statistical significance in a grouped analysis of relative expression (RE) normalized to OVA-CD8-DC (Fig. 2B). CD11b^+^ cDC subset of PLP-CD8-DC displayed a significant upregulation of CD40, CD83, CD86, PD-L2, and LAP and a higher frequency of CD86^+^ CD83^+^ and CD86^+^ PD-L2^+^ populations (Fig. 2C, Supplementary Figs. 4, 5). These findings suggest that PLP-CD8-DC progressively acquired a mature and regulatory phenotype following immunization.

### Interaction with PLP-CD8 in vitro influences cDC subsets towards a mature and regulatory phenotype

**Fig. 3:**
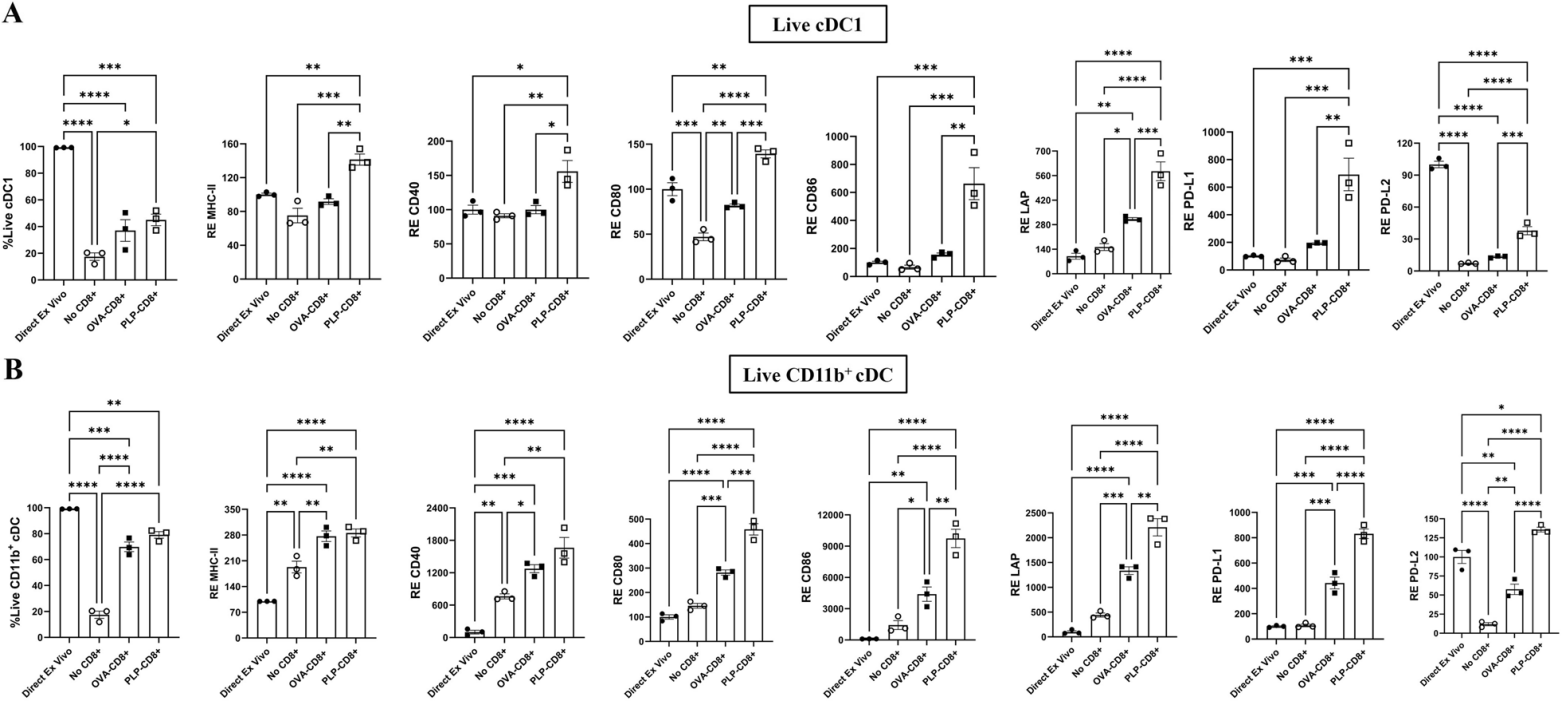
**PLP-CD8 promote cDC subsets to adopt a mature and regulatory phenotype in vitro.** Splenic DC from PLP-immunized mice were incubated with increasing ratios of PLP-CD8 or OVA-CD8 (1:1.25 to 1:5, DC: CD8^+^) in the presence of PLP_178-191_ (20 µg/ml) and rIL-2 (10 pg/ml) for 24 h. Freshly isolated DC (direct ex vivo) and DC incubated without CD8^+^ T cells (No CD8^+^) were used as controls. The figure shows the expression of costimulatory and regulatory markers on live cDC1 **A)** and CD11b^+^ cDC **B)** at DC: CD8, 1: 5. Marker expression was quantified as geometric mean fluorescence intensity (gMFI) and plotted as the relative expression (RE) normalized to the direct ex vivo condition. Interaction with PLP-CD8 enhanced the expression of costimulatory and regulatory markers on both cDC1 and CD11b^+^ cDC (N = 3, One-Way ANOVA with multiple comparisons, *p < 0.05, **p < 0.01, ***p < 0.001, ***p < 0.0001)

**Fig. 4:**
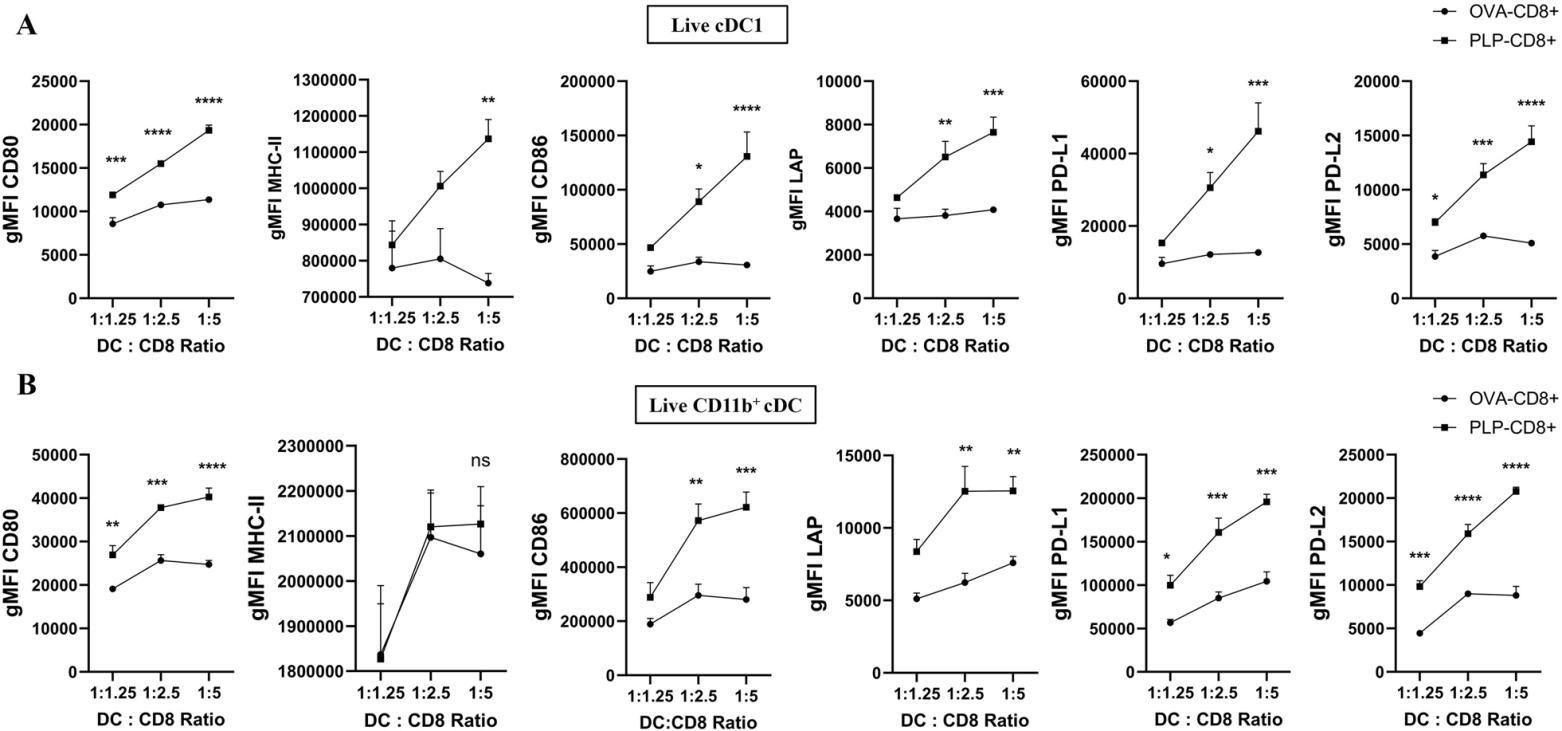
**PLP-CD8 influence cDC subsets in vitro in a dose-dependent manner. **Incubating DC with PLP-CD8 induced upregulation of costimulatory and regulatory markers on live **A)** cDC1 and **B)** CD11b^+^ cDC in proportion to the number of PLP-CD8 present in the condition (N = 3, Two-Way ANOVA, *p < 0.05, **p < 0.01, ***p < 0.001, ****p < 0.0001)

We hypothesized that the phenotypic changes observed in cDC subsets were driven by direct interaction with PLP-CD8. To test our hypothesis, bulk DC from PLP-immunized mice were cocultured with increasing ratios of PLP-CD8 or OVA-CD8 (DC:CD8 ratios ranging from 1:1.25 to 1:5) in the presence of PLP_178-191_ and rIL-2 for 24 h. Phenotypic changes in cDC subsets were assessed using flow cytometry, with freshly isolated DC (direct ex vivo) and DC incubated without CD8^+^ T cells (No CD8^+^) serving as controls. Marker expression was quantified in gMFI and normalized to the direct ex vivo condition. After 24 h, marked phenotypic changes were evident in both cDC subsets incubated with PLP-CD8, particularly at a DC:CD8 ratio of 1:5 (Fig. 3). Viability of both cDC subsets reduced drastically following incubation without CD8^+^ T cells and was significantly higher following incubation with either CD8^+^ T cells. Incubation with OVA-CD8 seemed to improve the expression of most markers on both cDC subsets than those without CD8^+^ T cells likely through non-specific mechanisms. cDC1 incubated with PLP-CD8 exhibited significantly higher levels of MHC-II, costimulatory markers (CD40, CD80, CD86), and regulatory markers (LAP, PD-L1, PD-L2), than with OVA-CD8 indicating an antigen-specific effect (Fig. 3A, Supplementary Figs. 6A, C). CD11b^+^ cDC incubated with PLP-CD8 showed similar upregulation of costimulatory and inhibitory markers, except for MHC-II, which was equally elevated in both PLP-CD8 and OVA-CD8 conditions (Fig. 3B, Supplementary Fig. 6B, D). Interestingly, the upregulation of costimulatory markers (excluding CD40) and regulatory markers in both cDC subsets, correlated with the number of PLP-CD8 present. Notably, PLP-CD8 led to a proportional increase in MHC-II expression on cDC1 but not cDC2 (Fig. 4). These findings suggest that PLP-CD8 influence both cDC subsets towards a mature and regulatory phenotype in an antigen-specific, dose-dependent manner.

Both cDC subsets present foreign antigens to CD4^+^ T cells [50]. cDC1 primarily drive Th1 differentiation while CD11b^+^ cDC mainly promote Th2 and Th17 lineages [51–53]. Importantly, cDC1 cross-present foreign antigens to CD8 + T cells to induce an effector response [54]. In turn, activated T cells enhance cDC survival to sustain immune responses, while regulatory CD4 + T cells (Tregs) suppress cDC activity to prevent excessive inflammation [41, 55]. The effects of interactions between activated CD8 + T cells and DC, particularly cDC1, remain poorly understood. Furthermore, the intercellular communication between cDC1 and CD11b^+^ cDC is also not well characterized. Based on the superiority of cDC1 cross-presentation ability, we hypothesized that PLP-CD8 predominantly engage with cDC1 and indirectly modulate CD11b^+^ cDC towards a mature, regulatory phenotype.

**Fig. 5:**
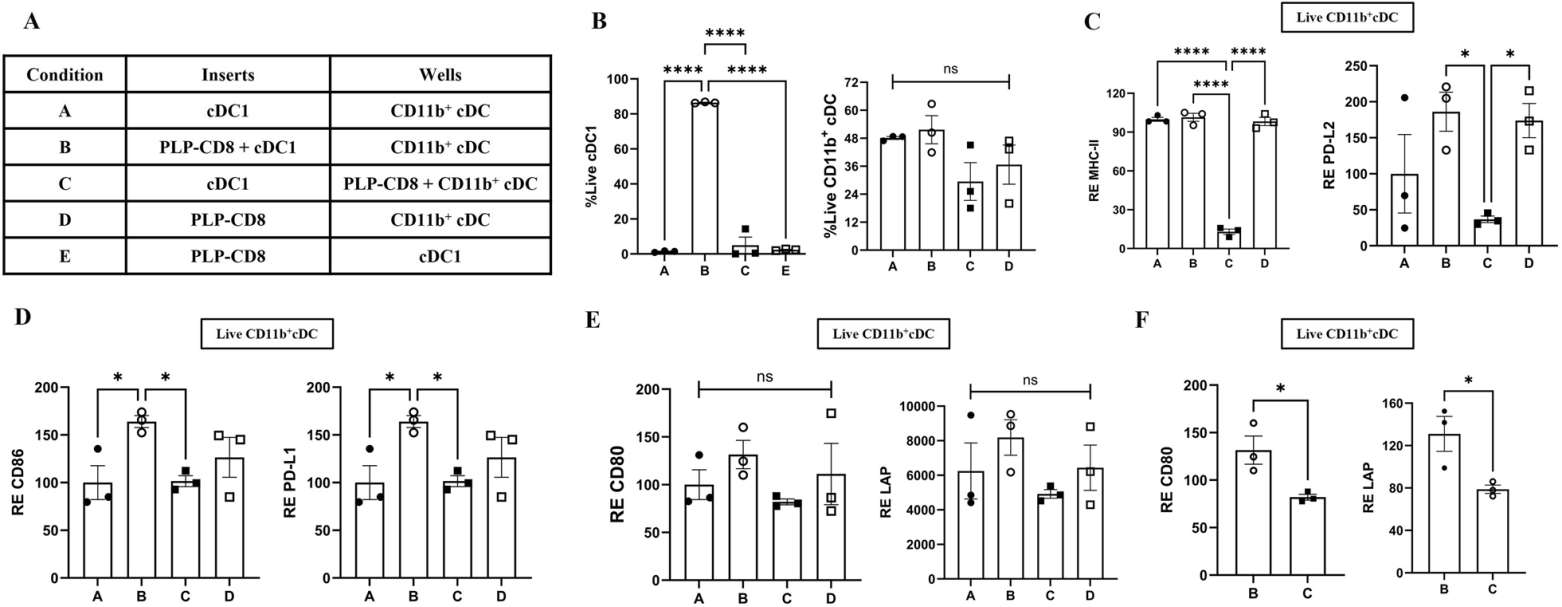
**Direct interaction between PLP-CD8 and cDC1 influences CD11b**^+^ **cDC via paracrine mechanisms**. cDC subsets, cDC1 and CD11b^+^ cDC were sorted using Fluorescence-Activated Cell Sorting (Cytek Aurora CS) from PLP-immunized mice 10 days post-immunization. For the trans-well assay, CD11b^+^ cDC (80,000) were seeded in the wells and cDC1 (20,000) into the inserts of the tissue-culture plate. PLP-CD8 (500,000) were added to either subset creating five conditions, shown in **A** Condition A: cDC1 in the inserts and CD11b^+^ cDC in the wells without PLP-CD8; Condition B: cDC1 and PLP-CD8 in the insert and CD11b^+^ cDC in the well; Condition C: cDC1 in the inserts and PLP-CD8 and CD11b^+^ cDC in the wells; Condition D: PLP-CD8 in the inserts and CD11b^+^ cDC in the wells without cDC1 and Condition E: PLP-CD8 in the inserts and cDC1 in the wells without CD11b^+^ cDC. The cells were incubated with PLP_178-191_ (20 µg/ml) and rIL-2 (10 pg/ml) for 24 h, following which, supernatants were collected, and cells were analyzed using flow cytometry. **B** Viable cDC1 were found only in Condition B (cDC1 in direct contact with PLP-CD8) whereas CD11b^+^ cDC viability was similar in all conditions. **C**-**F** Expression of indicated markers on live CD11b^+^ cDC was plotted as relative expression (RE) normalized to Condition A. **C)** Live CD11b^+^ cDC in Condition C (CD11b^+^ cDC in direct contact with PLP-CD8) displayed significant downregulation of MHC-II and PD-L2. **D** CD11b^+^ cDC in Condition B (cDC1 in direct contact with PLP-CD8) expressed significantly higher levels of CD86 and PD-L1. **E**–**F** CD11b^+^ cDC in Condition B displayed an increased expression of CD80 and LAP; however, this increase was not statistically significant when analyzed across all conditions using one-way ANOVA with multiple comparisons. Notably, a direct comparison between Conditions B and C using a t-test revealed statistical significance between the two conditions (N = 3, One-Way ANOVA with multiple comparisons, T-test, ns = not significant, *p < 0.05, **p < 0.01, ****p < 0.0001)

To investigate this hypothesis, we employed a trans-well assay system. FACS-sorted cDC1 and CD11b^+^ cDC subsets from PLP-immunized mice were seeded into the inserts and wells of a tissue-culture plate, respectively. PLP-CD8 were added to either subset, creating five experimental conditions: (A) cDC1 separated from CD11b^+^ cDC without PLP-CD8 (cDC1 **|** CD11b^+^ cDC); (B) PLP-CD8 with cDC1, separated from CD11b^+^ cDC (cDC1 + PLP-CD8 **|** CD11b^+^ cDC); (C) cDC1 separated from CD11b^+^ cDC with PLP-CD8 (cDC1 **|** CD11b^+^ cDC + PLP-CD8); (D) PLP-CD8 separated from CD11b^+^ cDC without cDC1 (PLP-CD8 **|** CD11b^+^ cDC); and (E) PLP-CD8 separated from cDC1 without CD11b^+^cDC (PLP-CD8 **|** cDC1) (Fig. 5A, Supplementary Fig. 7). Cells were incubated with PLP_178-191_ and rIL-2 for 24 h following which supernatants were collected and cells were analyzed using flow cytometry. Viability was assessed using Live-Dead blue and surface marker expression on live cells was quantified as gMFI and normalized to Condition A (cDC1 **|** CD11b^+^ cDC).

After 24 h of incubation, viable cDC1 were detected only in Condition B (cDC1 + PLP-CD8 **|** CD11b^+^ cDC) (Fig. 5B). We therefore excluded Condition E (PLP-CD8 **|** cDC1), which did not involve CD11b^+^ cDC, from further analysis. The viability of CD11b^+^ cDC showed slight variation across the various conditions, although these differences were not statistically significant (Fig. 5B).

**Fig. 6:**
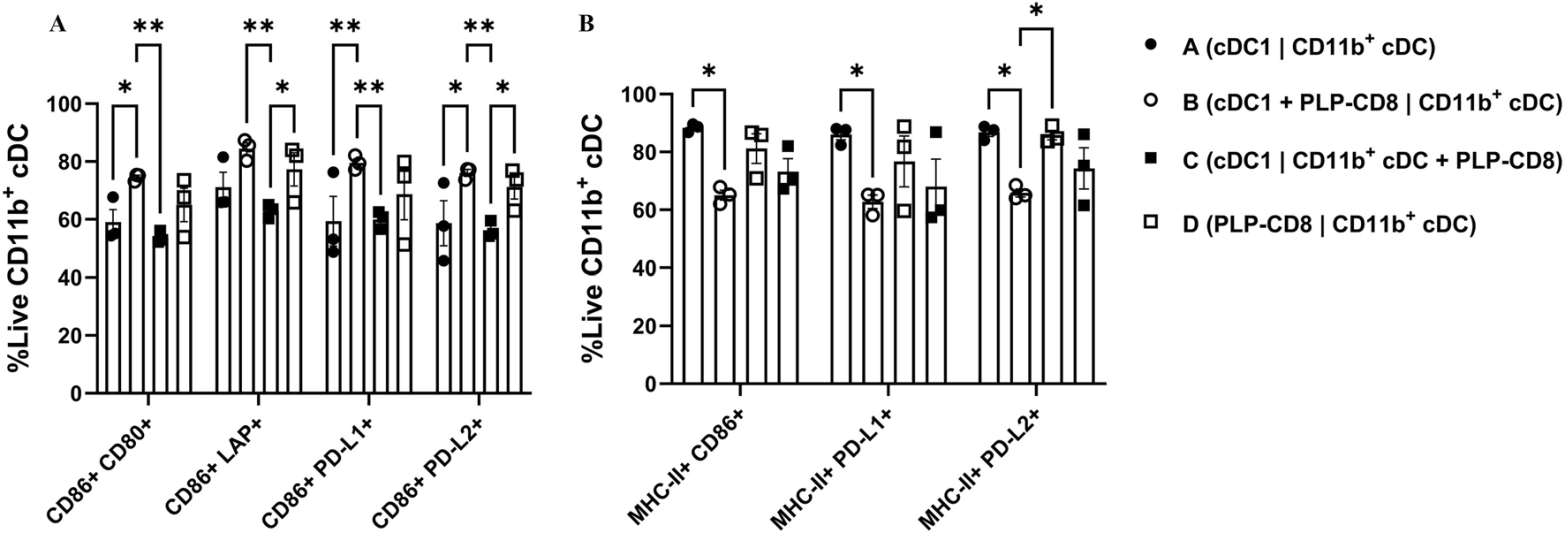
**Direct interaction between PLP-CD8 and cDC1 promotes co-expression of costimulatory and regulatory marker expression on CD11b**^+^ **cDC**. Co-expression of CD86 or MHC-II with other costimulatory and regulatory markers on live CD11b^+^ cDC was assessed using quadrant gating. CD11b^+^ cDC in Condition B (cDC1 in direct contact with PLP-CD8) displayed higher co-expression of **A** CD86 with CD80, LAP, PD-L1 and PD-L2, and of **B** MHC-II with CD86, PD-L1 and PD-L2 (N = 3, Two-Way ANOVA with multiple comparisons, *p < 0.05, **p < 0.01, ***p < 0.001, ****p < 0.0001)

In Condition B, where CD11b^+^ cDC sat across the transwell from cDC1 + PLP-CD8, they exhibited significant upregulation of CD86 and PD-L1 (Fig. 5D, Supplementary Fig. 8). They also expressed higher levels of CD80 and LAP, which were statistically significant in head-to-head comparisons (Fig. 5E, F). In the same condition, they also displayed a significantly higher frequency of populations co-expressing costimulatory and regulatory markers – CD86^+^ CD80^+^, CD86^+^ PD-L1^+^, CD86^+^ PD-L2^+^, MHC-II^+^ CD86^+^ and MHC-II^+^ PD-L1^+^ (Fig. 6, Supplementary Fig. 9). Of note, this phenotype matched that seen in our ex vivo analysis.

In contrast, in Condition C, where CD11b^+^ cDC directly interacted with PLP-CD8, they displayed significantly lower levels of MHC-II and PD-L2 than in other conditions (Fig. 5C, Supplementary Fig. 8). In Condition D (PLP-CD8 **|** CD11b^+^ cDC), CD11b^+^ cDC expressed most costimulatory and regulatory markers higher than in Condition C (cDC1 **|** CD11b^+^ cDC + PLP-CD8) (Figs. 5, 6; Supplementary Figs. 8, 9).

These results highlight the distinct effects of PLP-CD8 on cDC subsets. Direct contact with PLP-CD8 was necessary for the survival of cDC1 but not for CD11b^+^ cDC. Their interaction led to the upregulation of costimulatory and regulatory markers on CD11b^+^ cDC likely through paracrine mechanisms. In contrast, direct contact with PLP-CD8 had detrimental effects on CD11b^+^ cDC.

**Fig. 7:**
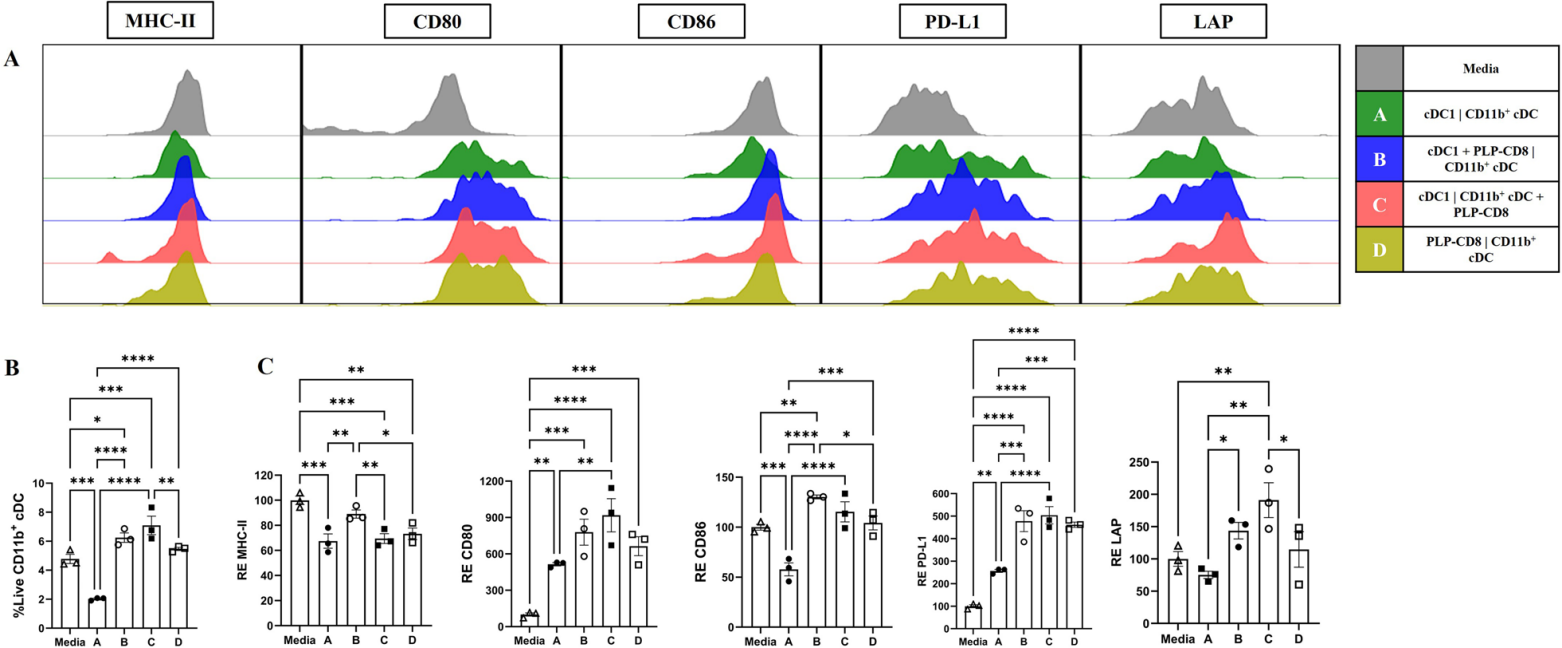
**PLP-CD8-conditioned supernatants promote survival, maturation, and regulatory marker expression in CD11b**^+^ **cDC**. CD11b^+^ cDC (45,000) were incubated with 500 µl of complete media or supernatants from the four conditions of the trans-well assay, sups A-D, for 24 h and analyzed using flow cytometry. **A** Shows the expression of costimulatory and regulatory markers on live CD11b^+^ cDC incubated with media or different supernatants. **B** Frequency of live CD11b^+^ cDC was significantly higher following incubation with media or supernatants from conditions involving PLP-CD8 (B-D). **C** Compares the expression of indicated markers plotted as relative expression (RE) normalized to media. Expression of costimulatory and regulatory markers on live CD11b.^+^ cDC was significantly higher following incubation with supernatants from conditions involving PLP-CD8 (**B**-**D**) (N = 3, One-Way ANOVA with multiple comparisons, *p < 0.05, **p < 0.01, ***p < 0.001, ****p < 0.0001)

To explore this further, CD11b^+^ cDC were incubated for 24 h with complete media or supernatants from the four trans-well conditions (Sups A-D). The viability of CD11b^+^ cDC was lowest with sup from Condition A (cDC1 **|** CD11b^+^ cDC) and highest with sup from Condition C (cDC1 **|** CD11b^+^ cDC + PLP-CD8) (Fig. 7B). CD11b^+^ cDC incubated with sup from Condition B (cDC1 + PLP-CD8 **|** CD11b^+^ cDC) displayed significantly higher levels of MHC-II than with sups from other conditions (Figs. 7A, C; Supplementary Fig. 10). CD11b^+^ cDC incubated with sup from Condition A (cDC1 **|** CD11b^+^ cDC) exhibited a lower expression of CD80, CD86, PD-L1 and LAP than with sups from other conditions (involving PLP-CD8) (Figs. 7A, C; Supplementary Fig. 10).

These findings show that the supernatants from all the conditions involving PLP-CD8 positively influenced CD11b^+^ cDC, unlike the direct interaction between the two cell types, suggesting potential paracrine mechanisms. Importantly, the phenotypic changes in CD11b^+^ cDC in earlier ex vivo and in vitro observations were similar to those induced in an indirect, paracrine fashion in the trans-well experiments. This suggests that, given the superiority of cDC1 at cross-presentation, in such conditions, PLP-CD8 may preferentially interact with cDC1 and subsequently modulate CD11b^+^ cDC using paracrine mechanisms.

### PLP-CD8-DC reveal transcriptomic changes in cDC subsets

**Fig. 8:**
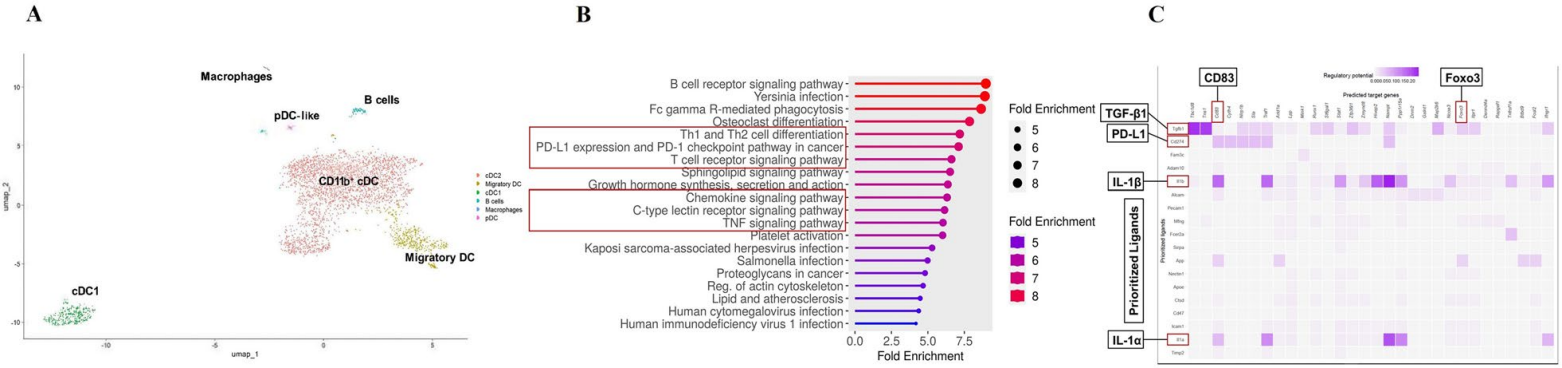
**Transcriptomic changes in cDC subsets influenced by adoptively transferred PLP-CD8.** PLP-CD8-DC and OVA-CD8-DC isolated on day 12 post-EAE induction were prepared for single-cell RNA sequencing (Parse Biosciences). Data were analyzed using the Seurat package in R. **A** Unsupervised clustering was performed, and clusters were annotated using canonical marker expression. **B** KEGG pathway analysis using upregulated genes in the CD11b^+^ cDC subset of PLP-CD8-DC showed enrichment of pathways concerning PD-1/PD-L1, Th1 & Th2 differentiation and chemokine and cytokine signaling. **C** Nichenet analysis identified prioritized ligands for potential intercellular communication between CD11b^+^ cDC and other DC subsets and non-DC antigen-presenting cells

To investigate the molecular basis of the regulatory changes, we performed single-cell RNA sequencing on splenic DC isolated from PLP-CD8 and OVA-CD8 recipients on day 12 post-EAE induction. Data were analyzed using the Seurat package in RStudio. Unsupervised clustering identified 9 distinct clusters (Supplementary Fig. 11A). Cell types were assigned based on canonical gene expression profiles where clusters were combined into 6 annotated groups (Fig. 8A, Supplementary Figs. 11B). Clusters 0, 1, 2, 3, and 6 exhibited similar gene expression patterns consistent with cDC2 or related DC subsets, characterized by the expression of CD11b (*Itgam*) and SIRP-α (*Sirpa*). Clusters 3 and 6 were designated as migratory cDC due to their expression of *Ccr7*. The remaining four clusters in this group lacked CD24 (*Cd24a*), CD64 (*Fcgr1*), and CD88 (*C5ar1*) but expressed CD26 (*Dpp4*), making monocyte-derived DC (moDC) or inflammatory DC unlikely and supporting their classification as cDC2. For clarity and convenience, we collectively refer to this group as CD11b⁺ cDC (Fig. 8A, Supplementary Figs. 11A, B). Differential gene expression (DGE) was analyzed within each DC subset from PLP-CD8 recipients compared to OVA-CD8 recipients. We found 168 significantly upregulated and 4 downregulated genes in CD11b^+^ cDC and 39 upregulated and 1 downregulated gene in the cDC1 subset from PLP-CD8 recipients (Supplementary Fig. 12A). Upregulated genes in CD11b^+^ cDC included key immunoregulatory genes such as TNF-receptor genes, *Cd274* (PD-L1), *Cd83*, *Mtor* and *Tgfb1* (TGF-β1) (Supplementary Fig. 12A)*.* KEGG analysis [47] of upregulated genes identified pathways concerning PD-1/PD-L1 interactions, Th1/Th2 differentiation and chemokine and cytokine signaling such as JAK2/STAT1 and PI3K/AKT signaling (Fig. 8B). NicheNet analysis [48] was used to model intercellular communication between CD11b^+^ cDC and other cell types. Top prioritized ligands were identified from all cell types whose receptor and target genes were upregulated in the CD11b^+^ cDC subset from PLP-CD8 recipients (Fig. 8C). Heatmaps and Circos plots [56] were used to visualize the predicted ligand-receptor and ligand-target interactions. The top prioritized ligands included *Tgfb1* (TGF-β1), *Cd274* (PD-L1), *Il1a* (IL-1α), and *Il1b* (IL-1β), with target genes such as *Cd83* and *Foxo3*, involved in immune regulation (Supplementary Fig. 12B). We used the decoupleR package [49] to assess transcription factor activity, revealing significantly higher *Foxo3* expression in both cDC1 and CD11b^+^ cDC subsets from PLP-CD8 recipients (Supplementary Fig. 12C). These transcriptomic findings align with our experimental results and offer new insights into the mechanisms of immune regulation mediated by myelin-reactive CD8^+^ T cells and splenic DC.

## Discussion

Myelin-reactive CD8^+^ T cells play a pivotal role in multiple sclerosis (MS) and experimental autoimmune encephalomyelitis (EAE) [6, 57]. Their adoptive transfer has been shown to inhibit EAE onset and mitigate disease severity in recipient mice in an antigen-specific, MHC-I-dependent manner [14, 21, 58]. We have demonstrated that adoptively transferred MOG_35-55_ reactive CD8^+^ T cells induced functional changes in the splenic dendritic cells (DC) of the recipient mice immunized with MOG_35-55_, but not in unimmunized recipients or those immunized with OVA_323-339,_ suggesting an antigen-specific effect [26]. We thus hypothesized that myelin-reactive CD8^+^ T cells induce regulatory DC in recipient mice, which subsequently suppress pathogenic CD4^+^ T cells and prevent EAE induction. To test our hypothesis, we analyzed the functional and phenotypic changes in conventional DC (cDC) subsets induced by CD8^+^ T cells reactive against PLP_178-191_ (PLP-CD8).

Adoptively transferred PLP-CD8 prevented EAE induction in recipient mice as shown before. This protection was associated with a reduced capacity of PLP-CD8-modulated DC (PLP-CD8-DC) to support CD4^+^ T cell proliferation and an altered cytokine profile characterized by reduced IL-12p70 and elevated IL-10 levels. These findings align with our previous studies [26] as well as others demonstrating the role of DC-derived cytokines in modulating T-cell responses [59–63].

Flow cytometric analysis revealed distinct phenotypic changes in both conventional DC (cDC) subsets from PLP-CD8 recipients.

Upregulation of PD-L2 was the earliest phenotypic change observed in both cDC subsets by day 6 post-EAE induction. By day 12, additional phenotypic changes became evident, with cDC1 maintaining PD-L2 upregulation, while CD11b^+^ cDC upregulated both costimulatory (CD40, CD83, CD86) and regulatory (PD-L2, LAP) markers, indicative of a mature and regulatory phenotype. This contrasts with the immature phenotype typically associated with regulatory DC generated in vitro or found naturally in vivo [64–66]. Instead, the observed phenotype closely resembles mature regulatory DC (Mreg) found in tumor microenvironments, known for their role in promoting immune tolerance to neoantigens [64–66].

Our in vitro experiments were designed to dissect the mechanisms underlying CD8^+^ T cell-mediated modulation of DC. Interaction with PLP-CD8 induced a dose-dependent upregulation of both costimulatory and regulatory markers on both cDC subsets corroborating our ex vivo findings. Notably, an increase in MHC-II expression was observed in cDC1 but not in CD11b^+^ cDC.

Using a trans-well assay, we demonstrated that direct contact with PLP-CD8 was essential for the survival of cDC1 but not CD11b^+^ cDC. Direct interaction of PLP-CD8 with cDC1 influenced CD11b^+^ cDC indirectly through paracrine signaling, promoting the expression of costimulatory and regulatory markers.

In contrast, direct contact between PLP-CD8 and CD11b^+^ cDC led to a marked reduction in the expression of MHC-II and PD-L2. These effects were contact-dependent, since supernatants from this condition did not replicate this phenotype, instead promoting a mature and regulatory phenotype in CD11b^+^ cDC. Furthermore, supernatants from the condition involving direct interaction between PLP-CD8 and cDC1 induced significant upregulation of MHC-II on live CD11b^+^ cDC. Interestingly, the contact-dependent negative effects of PLP-CD8 on CD11b^+^ cDC were absent when PLP-CD8 interacted with bulk, unsorted DC. Overall, this is in keeping with the model that PLP-CD8 might preferentially engage with cDC1, given their superiority for cross-presentation via MHC-I, and this interaction indirectly influenced CD11b^+^ cDC towards a mature and regulatory phenotype.

Single-cell RNA sequencing of PLP-CD8-DC versus OVA-CD8-DC revealed differentially expressed genes, including key immunoregulatory genes such as TNF-receptor genes, *Cd274* (PD-L1), *Cd83*, *Mtor* and *Tgfb1* (TGF-β1), predominantly in CD11b^+^ cDC but also in the cDC1 subset. KEGG pathway analysis [47] highlighted the enrichment of pathways associated with PD-1/PD-L1 interactions, Th1/Th2 differentiation, and chemokine and cytokine signaling, including JAK2/STAT1 and PI3K/AKT pathways linked to immune regulation and T-cell differentiation. NicheNet analysis [48] identified top-ranked ligands from interacting cell types, with their receptors and target genes upregulated in CD11b^+^ cDC subset of PLP-CD8-DC. These ligands included *Tgfb1* (TGF-β1), *Cd274* (PD-L1), *Il1a* (IL-1α), and *Il1b* (IL-1β), while their target genes such as *Cd83* and *Foxo3*, were implicated in immune regulation. These findings suggest a coordinated immune-regulatory network mediated by intercellular communication among DC subsets and other antigen-presenting cells. DecoupleR analysis [49] further highlighted the transcription factor, *Foxo3* upregulated in both cDC subsets. *Foxo3* is associated with regulatory DC and its deficiency may cause excessive T-cell proliferation and autoimmunity [67].

The bidirectional interaction between DC and T cells is well established [68]. However, the influence of CD8^+^ T cells on DC subsets, particularly on cDC1, remains poorly understood. Our results indicate that PLP-CD8 modulate cDC1 to indirectly influence CD11b^+^ cDC towards a regulatory phenotype, providing a potential mechanism for immune tolerance and modulation of pathogenic CD4^+^+ T cell responses. Further research is required to elucidate the precise mechanisms underlying these interactions and to explore their potential for developing targeted immunotherapies.

## Supplementary Information

Additional file 1.

## Data Availability

All pertinent data are included in this article, except for single-cell RNA-sequencing data, which we have deposited in NCBI’s Gene Expression Omnibus (GEO Series accession number GSE289845: https://www.ncbi.nlm.nih.gov/geo/query/acc.cgi?acc=GSE289845).

## Abbreviations

MS: Multiple sclerosis
EAE: Experimental autoimmune encephalomyelitis
APC: Antigen-presenting cells
DC: Dendritic cells
cDC: Conventional dendritic cells
cDC1 and CD11b^+^ cDC: Conventional DC subsets 1 and 2, respectively
PLP-CD8: In vitro-Activated enriched CD8^+^ T cells reactive against PLP_178-191_
OVA-CD8: In vitro-Activated enriched CD8^+^ T cells reactive against OVA_323-339_
PLP-CD8-DC: DC isolated from PLP-CD8 recipient mice
OVA-CD8-DC: DC isolated from OVA-CD8 recipient mice
Mregs: Mature, regulatory DC
CFSE: Carboxyfluorescein succinimidyl ester
