# Supplementary material for: Myelin-reactive CD8+ T cells influence conventional dendritic cell subsets towards a mature and regulatory phenotype in experimental autoimmune encephalomyelitis
Source: J Neuroinflammation. 2025 Feb 28;22:54. doi: 10.1186/s12974-025-03377-8 (PMC11869544; doi:10.1186/s12974-025-03377-8)
Supplement: Supplementary file 1 — Additional file 1. [file 12974_2025_3377_MOESM1_ESM.pdf]

Supplementary Figure 1: Timeline for adoptive CD8 transfer and isolation of splenic DC from CD8 recipients for further analysis

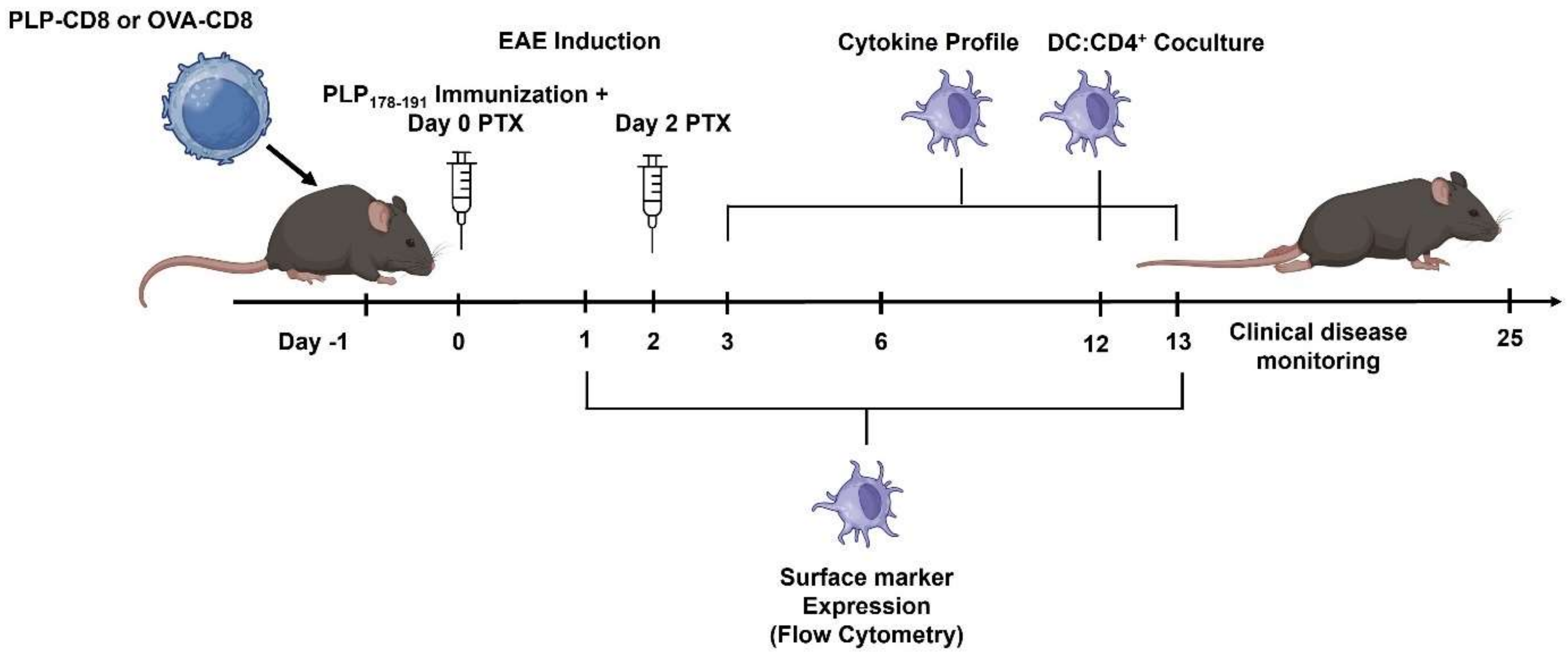

Supplementary Figure 1: Timeline for adoptive CD8 transfer and isolation of splenic DC from CD8 recipients for further analysis

Five million PLP-CD8 or OVA-CD8 were adoptively transferred into wild-type recipient mice on day -1. Mice were immunized with $PLP_{178\text{-}191}$ (100 µg) on day 0 and were given two doses of pertussis toxin (PTX, 250 ng) on days 0 and 2 to induce EAE. Splenic DC were isolated from PLP-CD8 recipients (PLP-CD8-DC) or OVA-CD8 recipients (OVA-CD8-DC) on designated time-points post-EAE induction for further analysis. DC were isolated on days 1, 3, 6 and 13 for phenotypic characterization by flow cytometry, on days 3 and 13 for the assessment of their cytokine profile and on day 12 for evaluating their capacity to support $CD4^{+}$ T cell proliferation.

Supplementary Figure 2: PLP-CD8-DC demonstrate reduced capacity to support CD4+ T cell proliferation

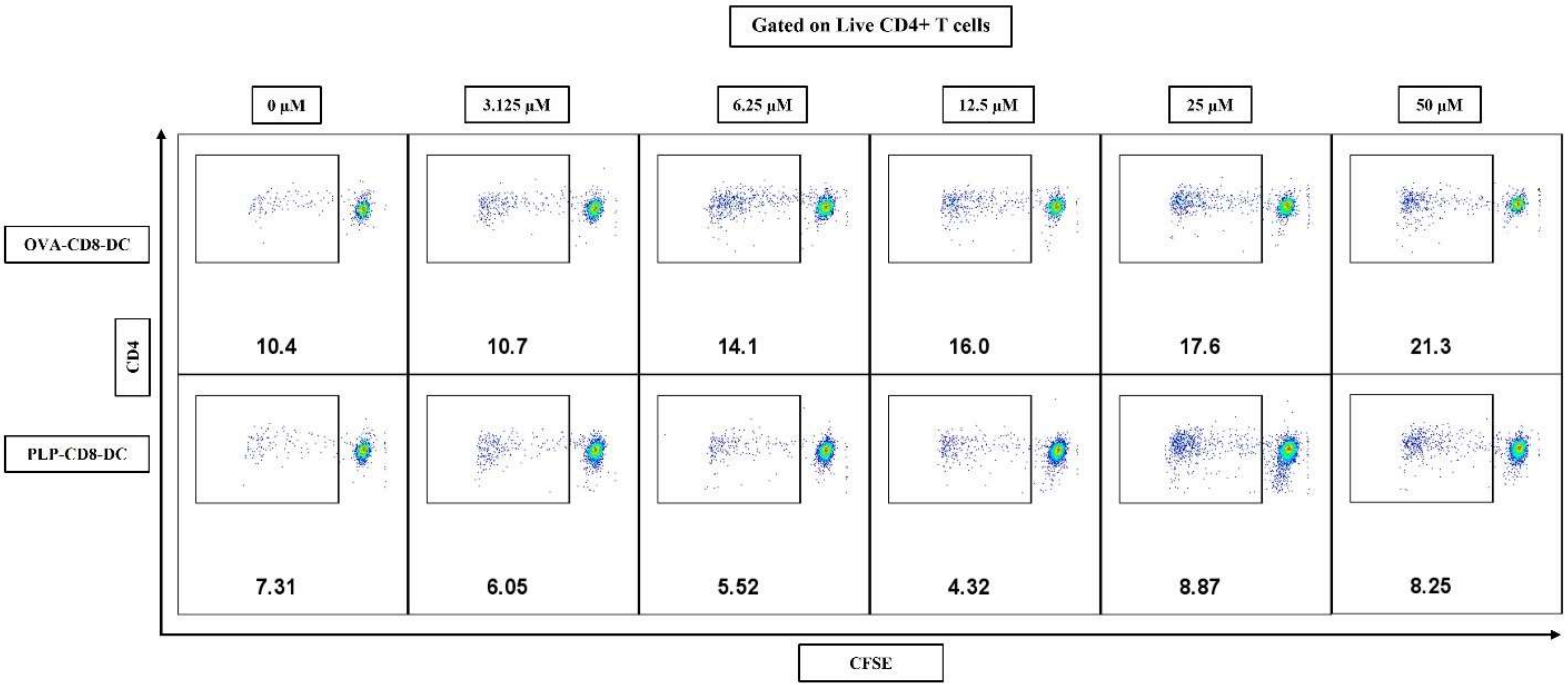

Supplementary Figure 2: PLP-CD8-DC demonstrate reduced capacity to support CD4+ T cell proliferation

Five million PLP-CD8 or OVA-CD8 were adoptively transferred into wild-type recipient mice on day -1. Mice were immunized with $PLP_{178-191}$ (100 µg) on day 0 and were given two doses of pertussis toxin (PTX, 250 ng) on days 0 and 2 to induce EAE. Splenic DC were isolated from PLP-CD8 recipients (PLP-CD8-DC) or OVA-CD8 recipients (OVA-CD8-DC) on day 12 post-EAE induction and cultured with CFSE-labelled responder $CD4^+$ T cells from PLP-immunized mice at a 1:10 ratio (DC: $CD4^+$) with increasing concentrations of $PLP_{178-191}$ (0 to 50 µM) for 5 days. Cells were stained with Live-Dead Blue and surface marker and analyzed using flow cytometry. $CD4^+$ T cell proliferation was quantified as the CFSE dilute fraction of live $CD4^+$ T cells. Representative pseudocolor plots are shown in the figure.

Supplementary Figure 3: Gating strategy for the phenotypic characterization of conventional DC subsets using flow cytometry

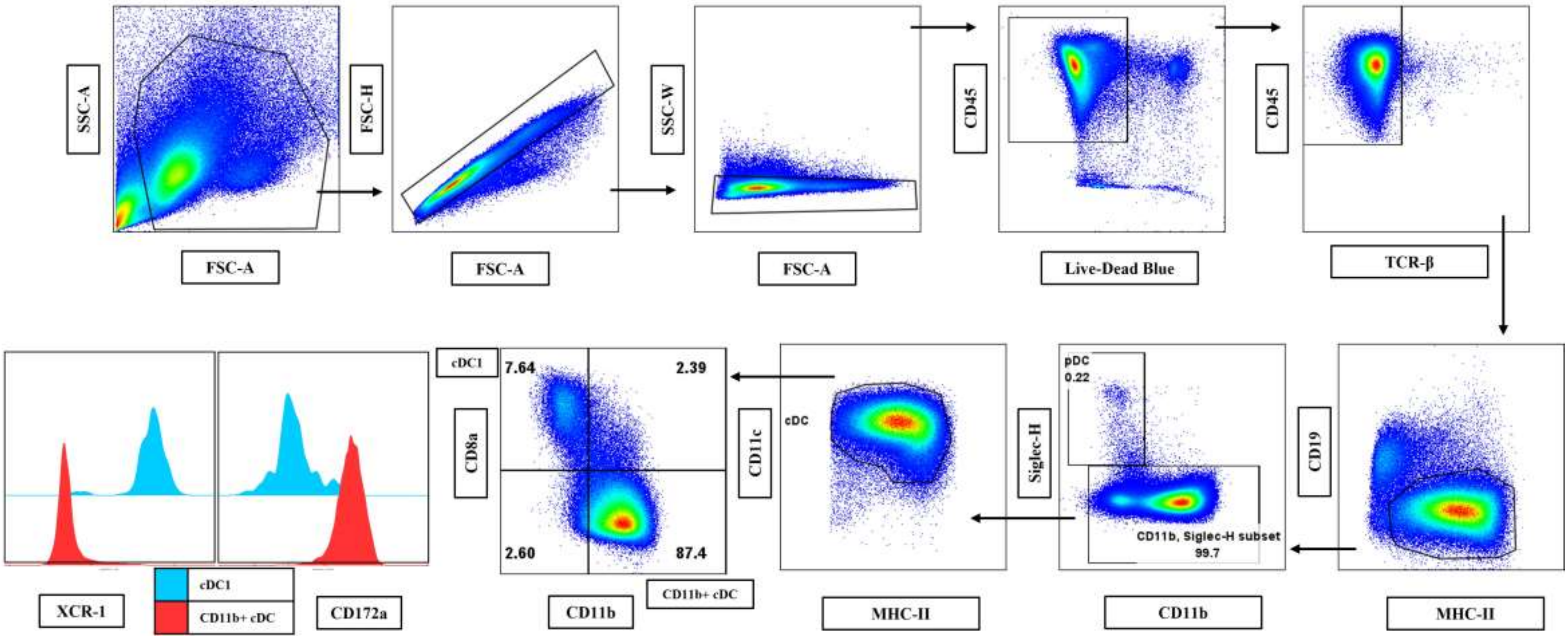

Supplementary Figure 3: Gating strategy for the phenotypic characterization of conventional DC subsets using flow cytometry

PLP-CD8-DC and OVA-CD8-DC were isolated on days 1, 3, 6 and 13 post-EAE induction and stained with Live-Dead Blue and surface markers for the phenotypic characterization of conventional DC (cDC) subsets using flow cytometry. After gating on Live CD45+ singlets; T cells, B cells and pDC were excluded and cDC were identified as $CD11c^{hi}$ $MHC\text{-}II^{hi}$. cDC subsets were further distinguished as cDC1 ($CD8a^{+}$ $CD11b^{-}$) and $CD11b^{+}$ cDC ($CD8a^{-}$ $CD11b^{+}$).

Supplementary Figure 4: PLP-CD8-DC subsets express higher levels of costimulatory and regulatory markers on day 13 post-EAE induction

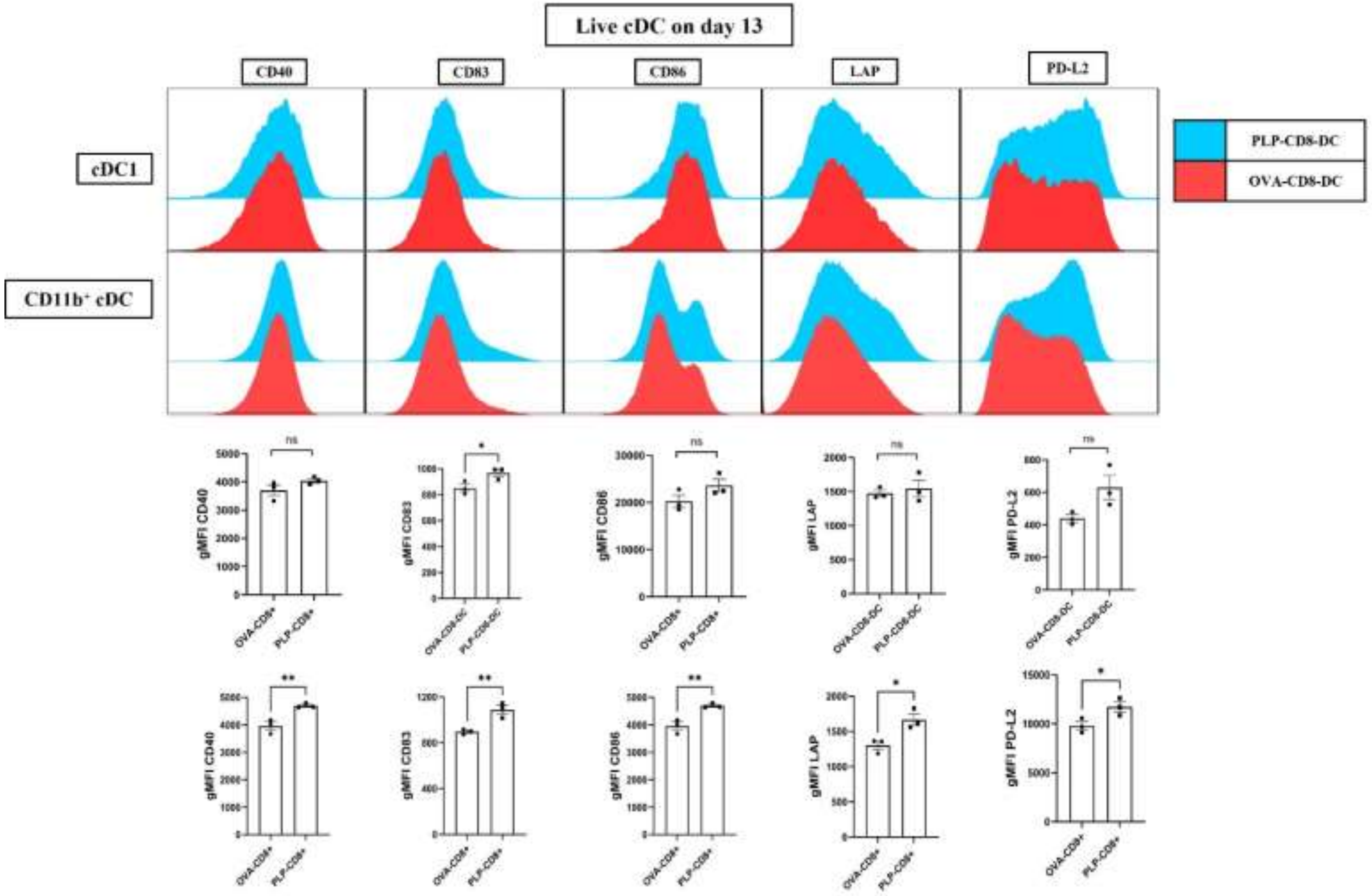

Supplementary Figure 4: PLP-CD8-DC subsets express higher levels of costimulatory and regulatory markers on day 13 post-EAE induction

Expression of the costimulatory and regulatory markers on live cDC1 and CD11b$^+$cDC isolated on day 13 post-EAE induction was measured in geometric mean fluorescence intensity (gMFI). The cDC1 and CD11b$^+$ cDC subsets of PLP-CD8-DC displayed higher expression of CD83 and of CD40, CD83, CD86, LAP and PD-L2, than OVA-CD8-DC, respectively (N = 3, T-test, ns = not significant, $*p < 0.05$, $**p < 0.01$).

Supplementary Figure 5: PLP-CD8-DC subsets exhibit higher coexpression of costimulatory and regulatory markers on day 13 post-EAE induction

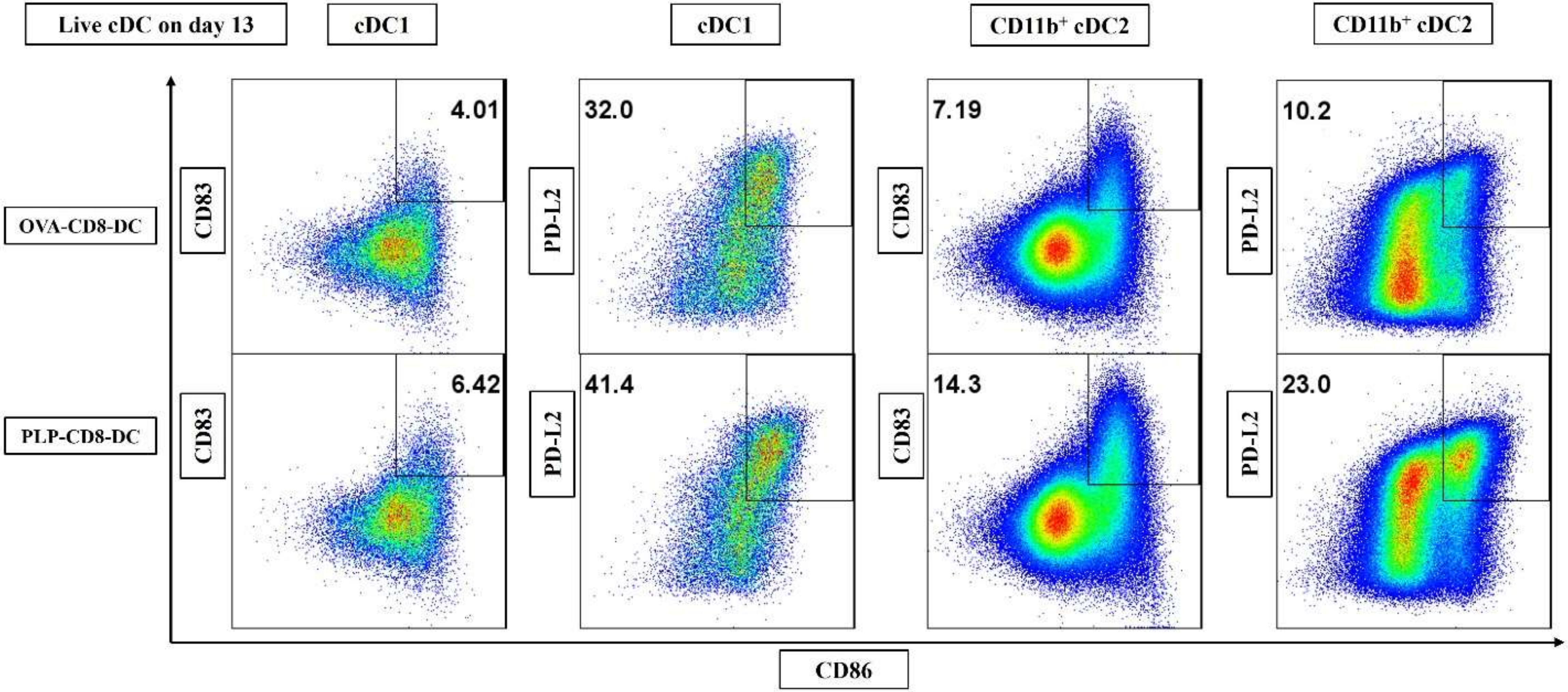

Supplementary Figure 5: PLP-CD8-DC subsets exhibit higher co-expression of costimulatory and regulatory markers on day 13 post-EAE induction

The representative pseudocolor plots demonstrate the co-expression of CD86 with CD83 and PD-L2 on live cDC1 and CD11b+ cDC isolated on day 13 post-EAE induction.

Supplementary Figure 6: PLP-CD8 enhance maturation and regulatory marker expression on cDC subsets *in vitro*

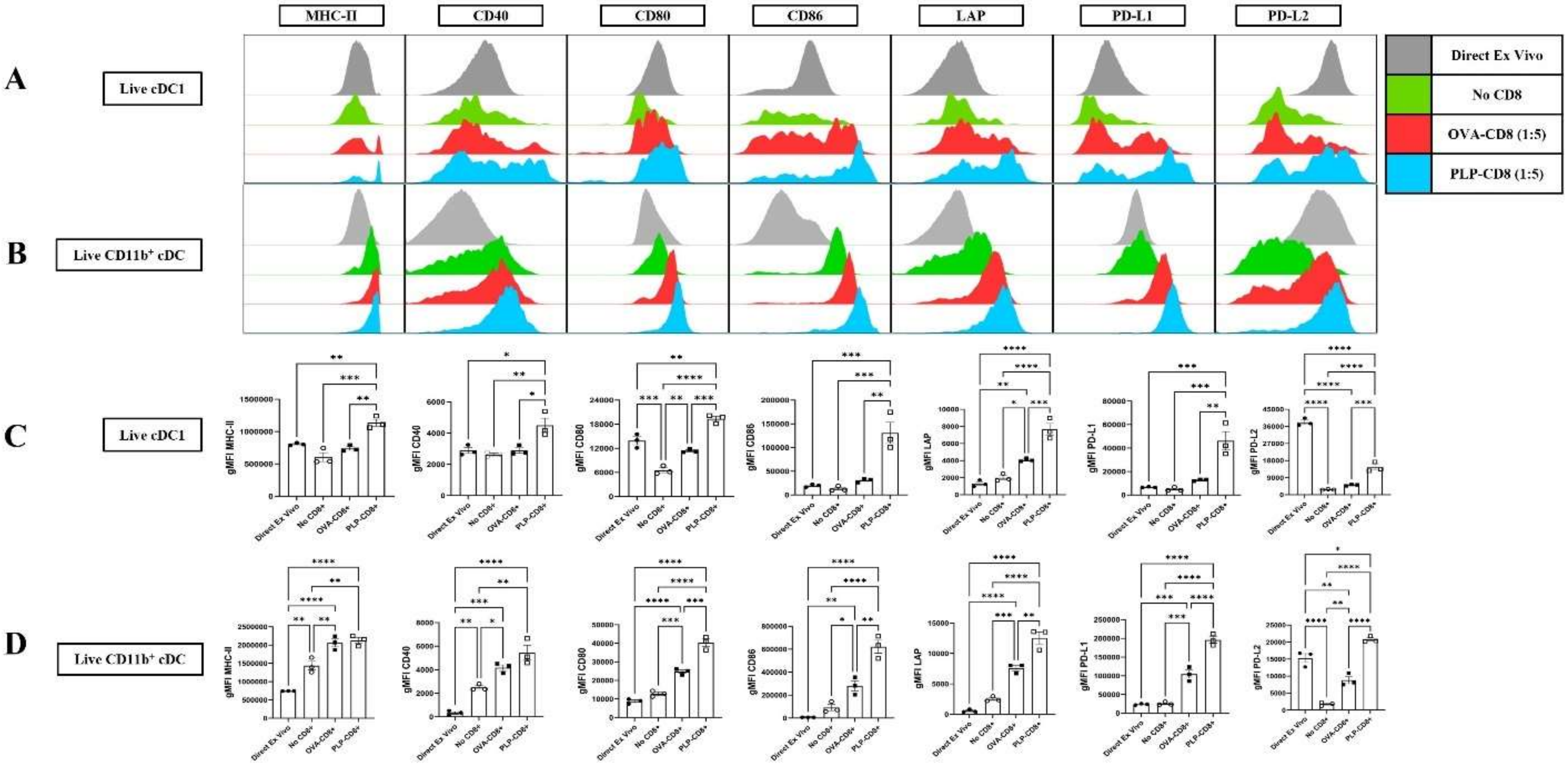

Supplementary Figure 6: PLP-CD8 enhance maturation and regulatory marker expression on cDC subsets *in vitro*

DC from PLP-immunized mice were cultured with PLP-CD8 or OVA-CD8 in increasing ratios (1:1.25 to 1:5, DC: CD8^{+}) in the presence of PLP$_{178-191}$ (20 µg/ml) and rIL-2 (10 pg/ml) for 24 hours. Freshly isolated DC (*Direct ex vivo*) and DC incubated without CD8^{+} T cells (No CD8^{+}) were used as controls. Following incubation, the expression of costimulatory and regulatory markers on live cDC1 and CD11b^{+} cDC subsets was measured in geometric mean fluorescence intensity (gMFI) using flow cytometry. Panels **A** and **B** show the expression of the indicated markers on live cDC1 and CD11b^{+} cDC, respectively, at a DC: CD8 ratio of 1:5. **C-D)** Expression of costimulatory and regulatory markers was significantly higher on live **C)** cDC1 and **D)** CD11b^{+} cDC following incubation with PLP-CD8 (N = 3, Owo-Way ANOVA with multiple comparisons, $*p < 0.05$, $**p < 0.01$, $***p < 0.001$, $****p < 0.0001$).

Supplementary Figure 7: Trans-well Assay: Experimental Setup

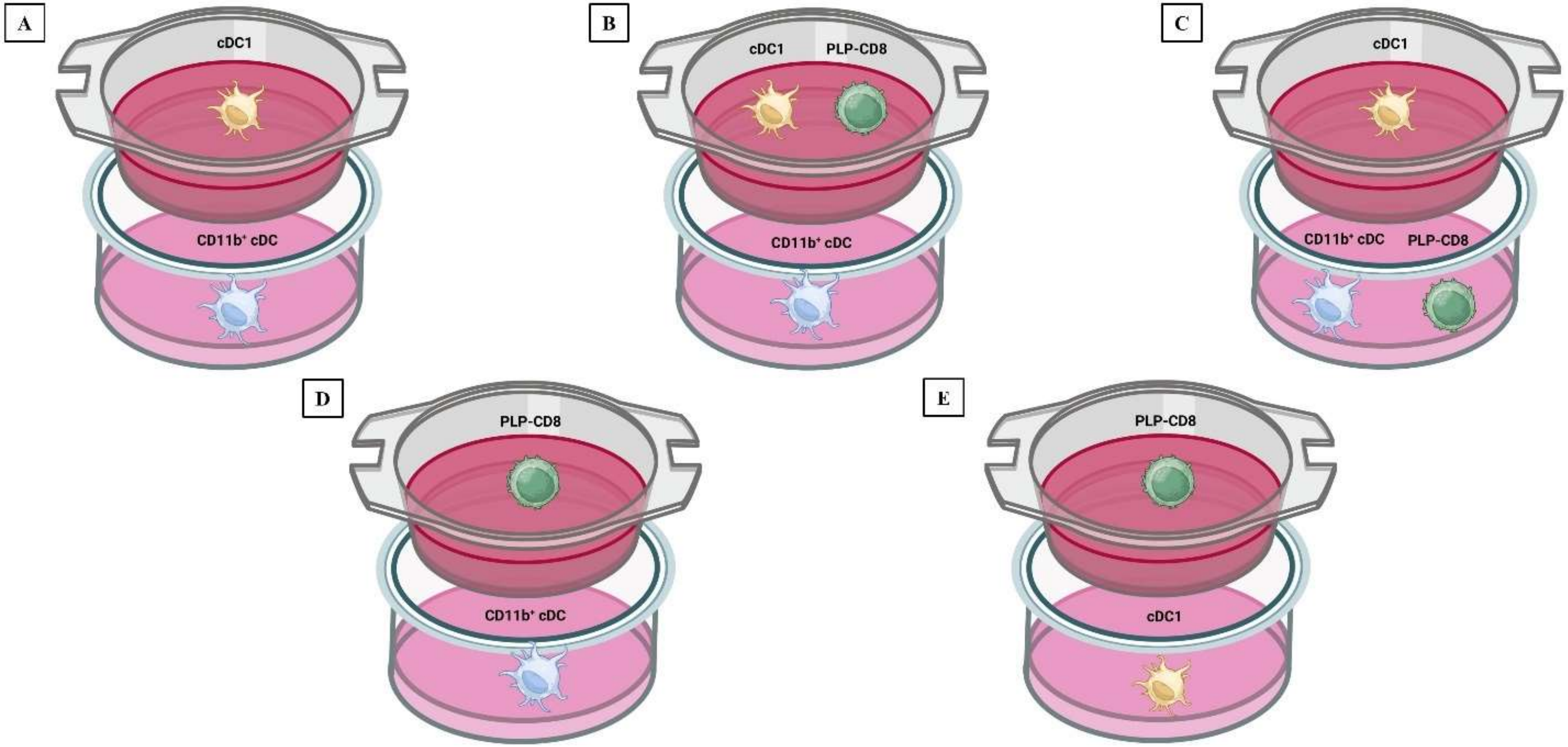

Supplementary Figure 7: Trans-well Assay: Experimental Setup

The interaction of PLP-CD8 with either cDC subset and its effect on the other subset were studied using a trans-well assay. cDC1 and $CD11b^+$ cDC, were FACS-sorted from PLP-immunized mice 10 days post-immunization. $CD11b^+$ cDC (80,000) were seeded into the wells and cDC1 (20,000) into the inserts of the tissue-culture plate. PLP-CD8 (500,000) were added to either subset creating five conditions: Condition **A**: cDC1 in the inserts and $CD11b^+$ cDC in the wells without PLP-CD8; Condition **B**: cDC1 and PLP-CD8 in the insert and $CD11b^+$ cDC in the well; Condition **C**: cDC1 in the inserts and PLP-CD8 and $CD11b^+$ cDC in the wells; Condition **D**: PLP-CD8 in the inserts and $CD11b^+$ cDC in the wells without cDC1; and Condition **E**: PLP-CD8 in the inserts and cDC1 in the wells without $CD11b^+$ cDC. The cells were incubated with $PLP_{178\text{-}191}$ (20 µg/ml) and rIL-2 (10 pg/ml) for 24 hours, following which, supernatants were collected, and cells were analyzed using flow cytometry. These illustrations were generated in BioRender.

Supplementary Figure 8: Paracrine effects of PLP-CD8 on CD11b^{+} cDC likely augmented by cDC1

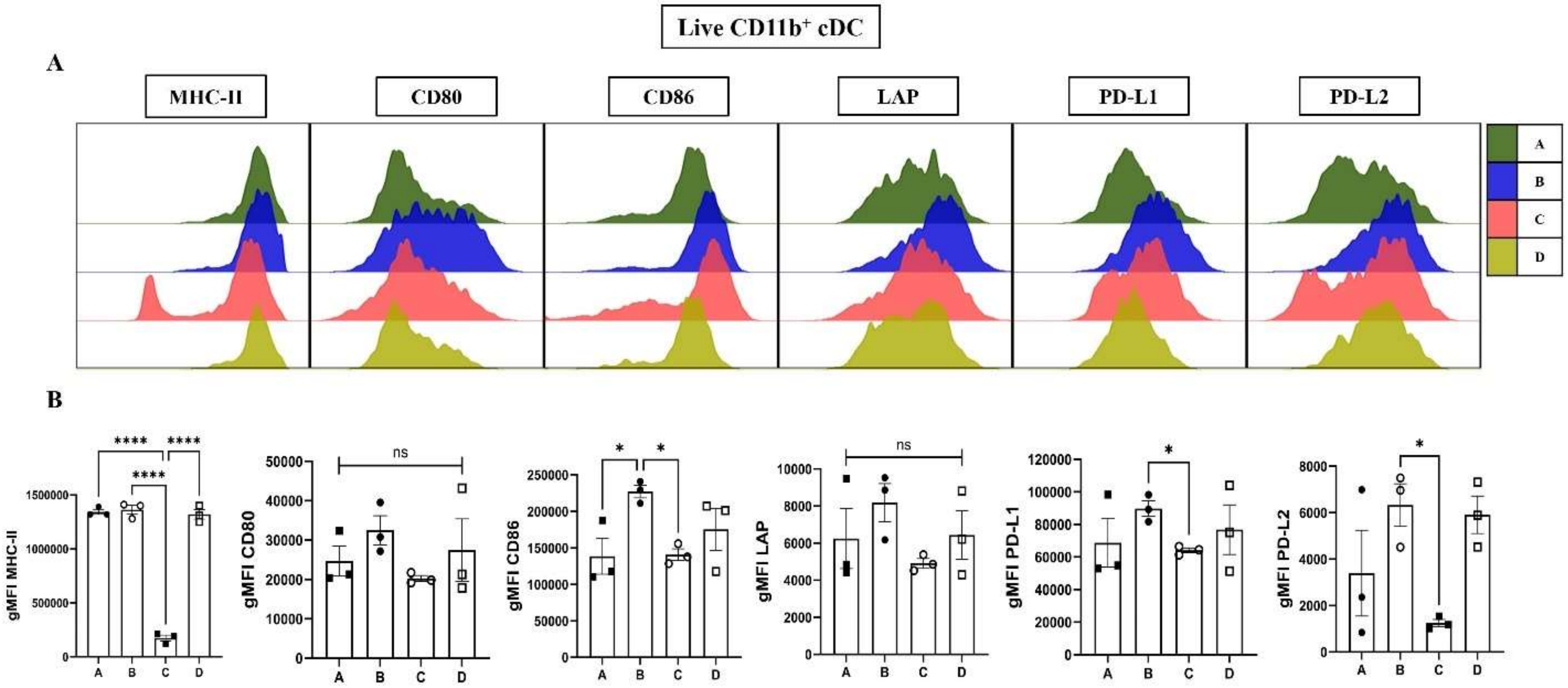

Supplementary Figure 8: Paracrine effects of PLP-CD8 on CD11b^{+} cDC likely augmented by cDC1

Following 24 hours of incubation in the trans-well assay, the expression of costimulatory and regulatory markers on live CD11b^{+} cDC was measured in geometric mean fluorescence intensity (gMFI). **A)** Shows the expression of these markers in different conditions. **B)** MHC-II expression on CD11b^{+} cDC was significantly reduced in Condition C (CD11b^{+} cDC in direct contact with PLP-CD8). CD11b^{+} cDC in Condition B (cDC1 in direct contact with PLP-CD8) displayed significantly higher expression of CD86 than in Condition A (cDC1 separated from CD11b^{+} cDC without PLP-CD8) and in Condition C. Expression of PD-L1 and PD-L2 on CD11b^{+} cDC was significantly higher in Condition B than in Condition C (N = 3, One-Way ANOVA with multiple comparisons, ns = not significant, *$p < 0.05$, ****$p < 0.0001$).

Supplementary Figure 9: Coexpression of costimulatory and regulatory molecules on live CD11b^{+} cDC in different trans-well conditions

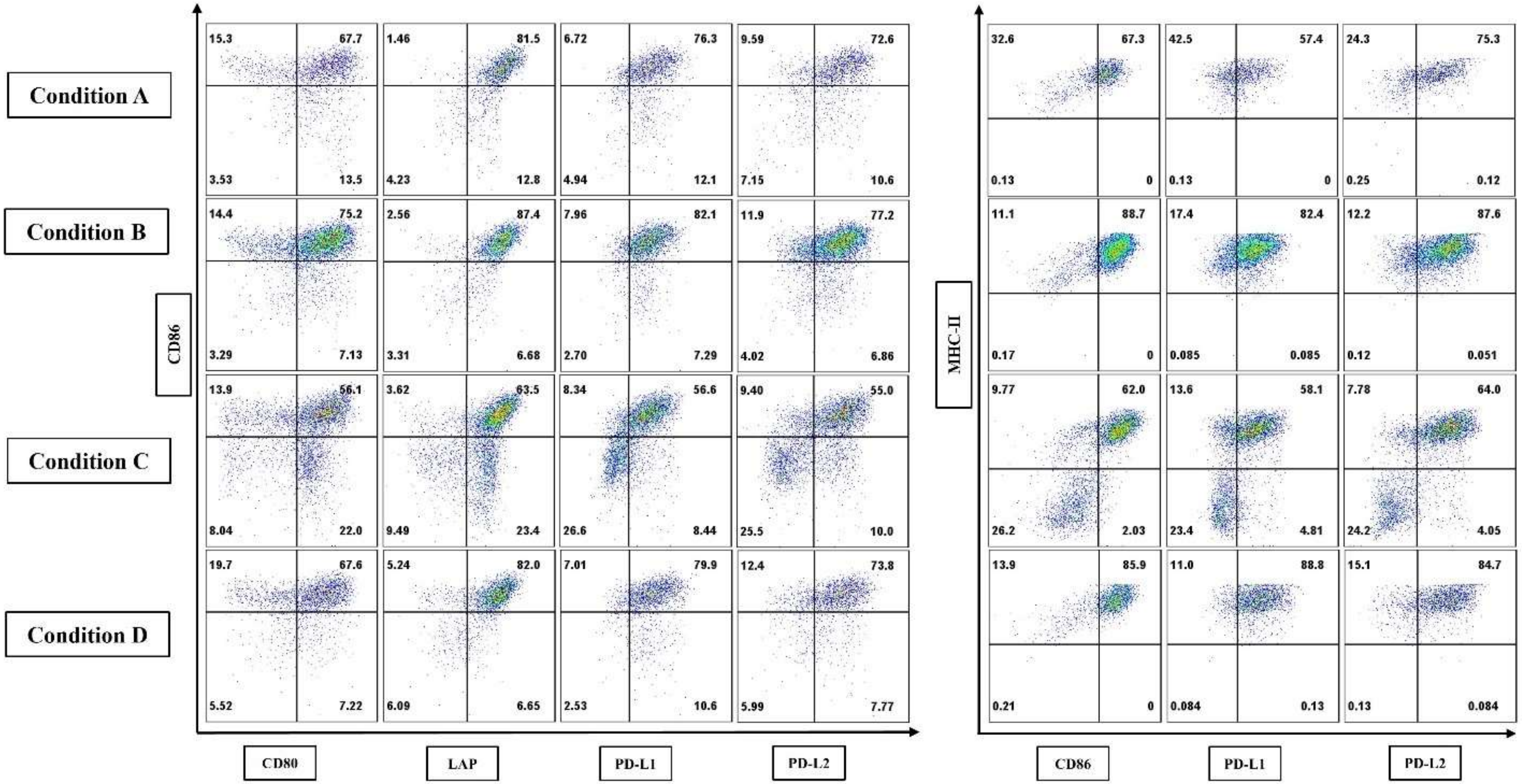

Supplementary Figure 9: Coexpression of costimulatory and regulatory molecules on live CD11b^{+} cDC in different trans-well conditions

This panel shows the co-expression of CD86 or MHC-II with other costimulatory and regulatory markers on live CD11b^{+} cDC in Conditions A-D in pseudocolor plots.

Supplemental Figure 10: PLP-CD8-conditioned supernatants promote survival, maturation, and regulatory marker expression in CD11b$^+$ cDC

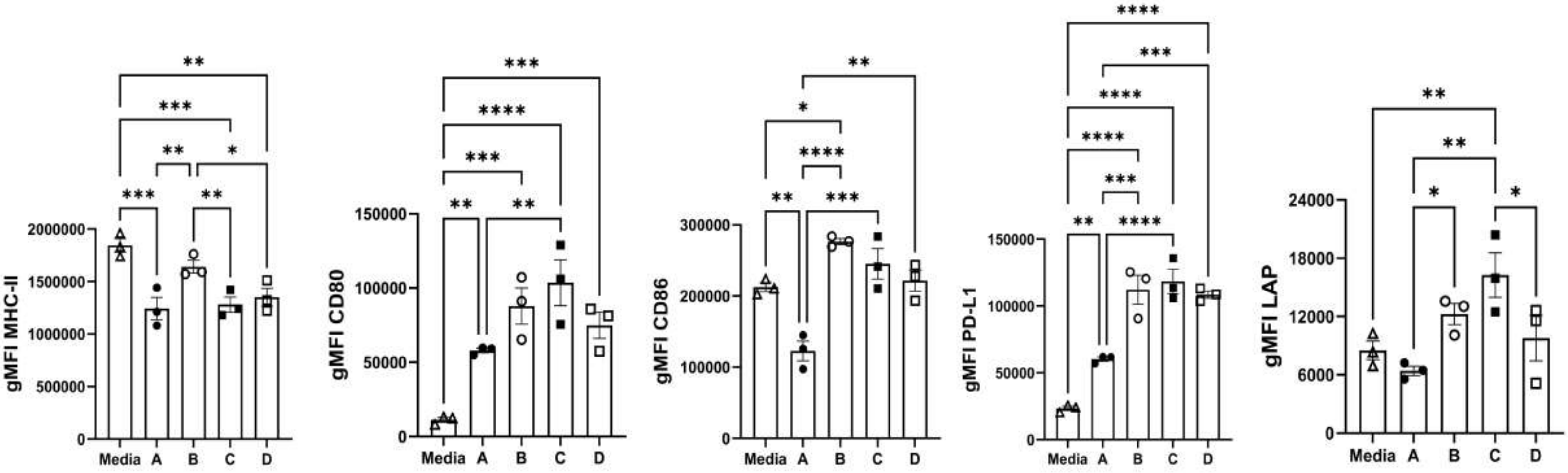

Supplemental Figure 10: PLP-CD8-conditioned supernatants promote survival, maturation, and regulatory marker expression in CD11b$^+$ cDC

CD11b$^+$ cDC (45,000) were incubated with 500 µl of complete media or supernatants from the four conditions of the trans-well assay, sups A-D, for 24 hours and analyzed using flow cytometry. Costimulatory and regulatory markers on live CD11b$^+$ cDC were quantified in geometric mean fluorescence intensity (gMFI). Incubation with Media had varying effects on the expression of different markers on CD11b$^+$ cDC. MHC-II expression on CD11b$^+$ cDC was highest when incubated with Media and was significantly higher with sup B (cDC1 in direct contact with PLP-CD8) than with other sups. In contrast, the expression of CD80, CD86, PD-L1 and PD-L2 on CD11b$^+$ cDC was lower with sup A (cDC1 separated from CD11b$^+$ cDC without PLP-CD8) than with other sups from conditions involving PLP-CD8 (N = 3, One-Way ANOVA with multiple comparisons, *$p < 0.05$, **$p < 0.01$, ***$p < 0.001$, ****$p < 0.0001$).

Supplementary Figure 11: Single-cell RNA-sequencing – Unsupervised clustering and cell type annotation

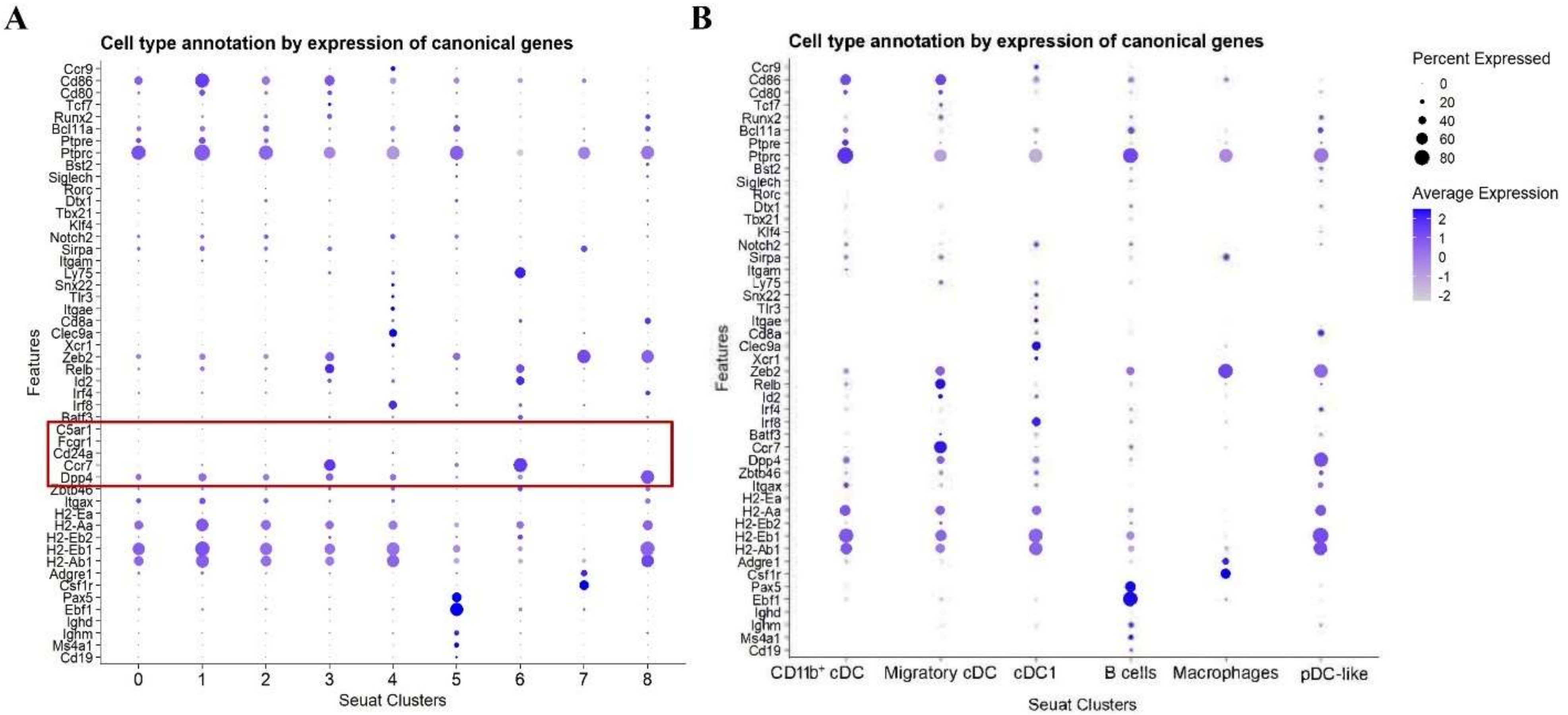

Supplementary Figure 11: Single-cell RNA-sequencing – Unsupervised clustering and cell type annotation

PLP-CD8-DC and OVA-CD8-DC isolated on day 12 post-EAE induction were prepared for single-cell RNA sequencing (Parse Biosciences). Data were analyzed using the Seurat package in R. Unsupervised clustering identified 9 distinct Seurat clusters. Canonical gene expression was used to designate different cell types. Panel **A)** shows the dot plot of canonical gene expression across 9 Seurat clusters while panel **B)** shows the dot plot of canonical gene expression in annotated Seurat clusters. Clusters 0, 1, 2, 3, and 6 exhibited similar gene expression patterns consistent with cDC2 or related DC subsets, characterized by the expression of CD11b (*Itgam*) and SIRP-α (*Sirpa*). Clusters 3 and 6 were designated as Migratory cDC due to their expression of *Ccr7*. The remaining four clusters in this group lacked CD24 (*Cd24a*), CD64 (*Fcgr1*), and CD88 (*C5ar1*) but expressed CD26 (*Dpp4*), making monocyte-derived DC (moDC) or inflammatory DC unlikely and supporting their classification as cDC2. For clarity and convenience, we collectively refer to this group as CD11b^{+} cDC.

Supplementary Figure 12: Single-cell RNA-sequencing – Differential gene expression (DGE) and downstream analysis

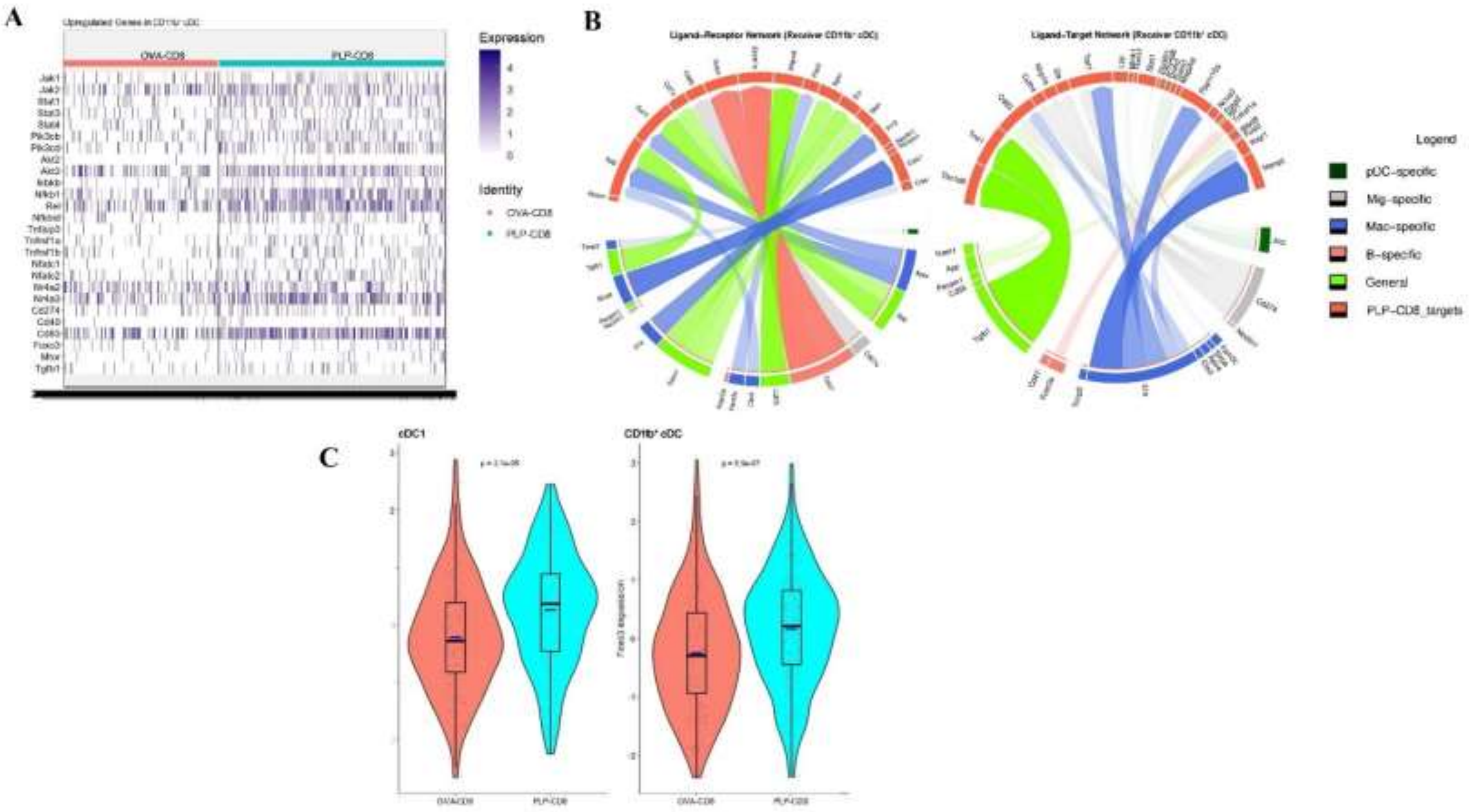

Supplementary Figure 12: Single-cell RNA-sequencing – Differential gene expression (DGE) and downstream analysis

Differential gene expression (DGE) was analyzed within each annotated cluster between the two CD8 treatments, PLP-CD8 and OVA-CD8. No significant DGE was observed in B cells, Macrophages, pDC-like cells and Migratory cDC. The highest number of differentially expressed genes were found in CD11b$^+$ cDC followed by cDC1. Panel **A)** shows a heatmap of representative genes upregulated in the CD11b$^+$ cDC subset from PLP-CD8 recipients. Nichnet analysis was performed to model potential intercellular communication between CD11b$^+$ cDC and other DC subsets and non-DC cell types. A list of prioritized ligands important for intercellular communication was generated from the dataset with their target and receptor genes upregulated in CD11b$^+$ cDC from PLP-CD8 recipients. Panel **B)** shows the ligand-receptor and ligand-target networks using Circos plots. Panel **C)** shows significant upregulation of *Foxo3*, a key immunoregulatory transcription factor in cDC1 and CD11b$^+$ cDC from PLP-CD8 recipients identified using DecoupleR analysis.
